# Direct Regeneration of Spent Lithium-Ion Battery Cathodes: From Theoretical Study to Production Practice

**DOI:** 10.1007/s40820-024-01434-0

**Published:** 2024-05-31

**Authors:** Meiting Huang, Mei Wang, Liming Yang, Zhihao Wang, Haoxuan Yu, Kechun Chen, Fei Han, Liang Chen, Chenxi Xu, Lihua Wang, Penghui Shao, Xubiao Luo

**Affiliations:** 1 National–Local Joint Engineering Research Center of Heavy Metals Pollutants Control and Resource Utilization, Nanchang Hangkong University, Nanchang, 330063 People’s Republic of China; 2 Key Laboratory of Hunan Province for Advanced Carbon–based Functional Materials, School of Chemistry and Chemical Engineering,, Hunan Institute of Science and Technology, Yueyang, 414006 People’s Republic of China; 3 College of Materials Science and Engineering, Central South University of Forestry and Technology, Changsha, 410004 People’s Republic of China; 4 School of Life Science, Jinggangshan University, Ji’an, 343009 People’s Republic of China

**Keywords:** Spent LIBs, Failure reasons, Cathode recycling, Direct regeneration, Production practice

## Abstract

Direct regeneration method has been widely concerned by researchers in the field of battery recycling because of its advantages of in situ regeneration, short process and less pollutant emission. In this review, we firstly analyze the primary causes for the failure of three representative battery cathodes (lithium iron phosphate, layered lithium transition metal oxide and lithium cobalt oxide), targeting at illustrating their underlying regeneration mechanism and applicability. Efficient stripping of material from the collector to obtain pure cathode material has become a first challenge in recycling, for which we report several pretreatment methods currently available for subsequent regeneration processes. We review and discuss emphatically the research progress of five direct regeneration methods, including solid-state sintering, hydrothermal, eutectic molten salt, electrochemical and chemical lithiation methods. Finally, the application of direct regeneration technology in production practice is introduced, the problems exposed at the early stage of the industrialization of direct regeneration technology are revealed, and the prospect of future large-scale commercial production is proposed. It is hoped that this review will give readers a comprehensive and basic understanding of direct regeneration methods for used lithium-ion batteries and promote the industrial application of direct regeneration technology.

## Highlights

- Analyze the primary causes of cathode failure in three representative batteries, illustrating their underlying regeneration mechanism.
- The latest research status of direct regeneration of spent lithium–ion batteries was reviewed and summarized in focus.
- The application examples of direct regeneration technology in production practice are introduced for the first time, and the problems exposed in the initial stage of industrialization were revealed.

## Graphical abstract

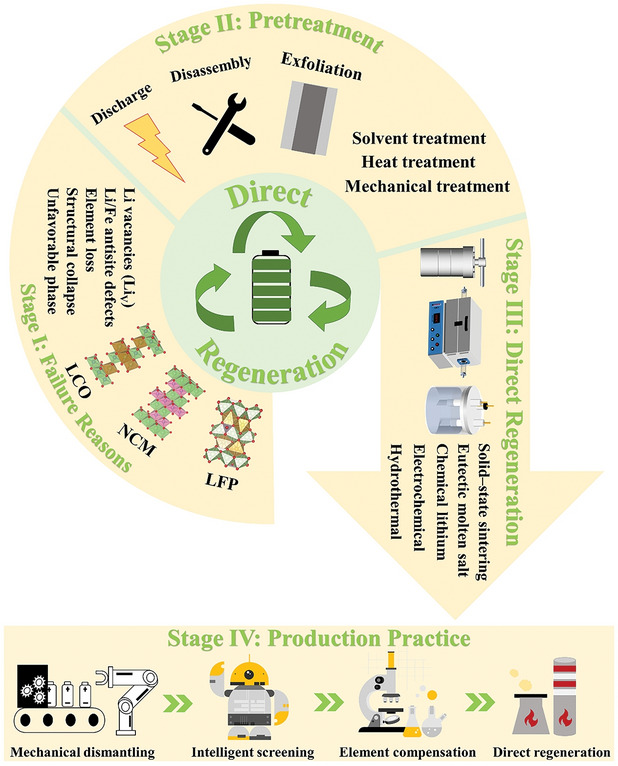

## Introduction

To alleviate the scarcity of fossil energy and decrease the reliance of fossil fuels, the development of new energy vehicles has been prospering in recent years [1–4]. This substantial increase in shipments will undoubtedly lead to a surge in the retirement of lithium-ion batteries (LIBs) in the near future [5–7]. Research reveals that LIBs contain a large number of valuable metal elements, such as lithium, nickel, cobalt, etc., which are mainly concentrated in the cathode materials [8–10]. The World Health Organization has classified the inner nickel and cobalt elements as carcinogens. The polyvinylidene fluoride (PVDF) binder can undergo decomposition at high temperature, resulting in the release of corrosive HF [11–13]. Furthermore, the organic electrolyte can hydrolyze upon exposure to moisture, and release toxic fluorine-containing compounds [14]. These factors contribute to severe pollution of soil, water and air. According to the estimate from U.S. Department of Energy, incorporating recycled materials from the used LIBs into the production of new batteries can result in a 40% reduction in costs, an 82% decrease in energy consumption and a 91% decrease in greenhouse gas emission, respectively [15–17]. Therefore, the effective recycling and reuse of spent LIBs materials is of utmost importance in mitigating or even resolving the energy/resource crisis and environment pollution.

**Fig. 1:**
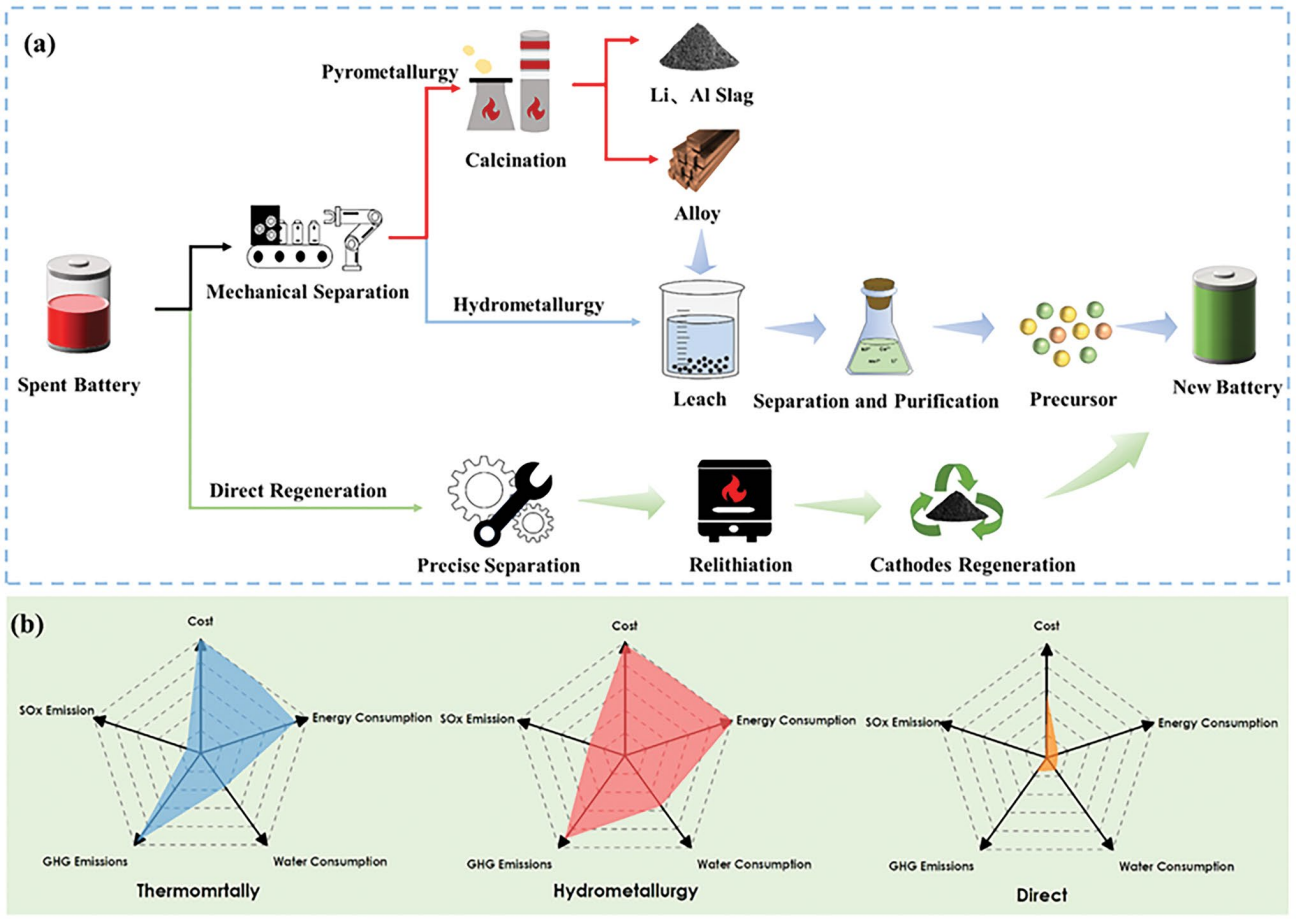
**a** Flow diagram of three mainstream methods for battery recycling: pyrometallurgy, hydrometallurgy and direct regeneration. **b** A comparison of three recycling methods to achieve the production of 1 kg of NMC111 cathode in terms of cost, energy consumption, water consumption, GHG emission and sulfur oxide emission [30]

Up to date, the mainstream methods for battery recycling include pyrometallurgy, hydrometallurgy and direct regeneration (Fig. 1a) [18]. Pyrometallurgy is considered as a scalable approach, and has been implemented in practical production [19, 20]. The spent batteries are mechanically disassembled and transferred to a furnace for high temperature (> 1000 °C) smelting, which exacerbates energy consumption to a certain extent as the whole recycling process needs to be carried out continuously at high temperatures. At the same time, electrolyte, carbon black, organic binder and other impurities are decomposed into HF, CO_2_ and other volatile gases. In order to avoid them spreading into the atmosphere, it is also needed to add the subsequent flue gas treatment device, which inadvertently increases the production costs of enterprises. Lithium metal is usually ignored in the form of slag, and other valuable metals are collected in the form of melt or alloy, leading to difficult separation process and low metal recovery rate [21–26]. In regards to hydrometallurgy, its primary objective is to convert the metals present in used batteries into metal ions through the assistance of acidic or alkaline solutions. The obtained metal ions are recovered through a series of steps such as precipitation, extraction and deposition, and finally are often involved in the process of battery regeneration in the form of precursors. The large use of acid and alkali reagents will cause secondary pollution to the environment, and the whole recycling process is time–consuming and highly expensive [27–29].

Compared with the above two recycling methods, direct regeneration with the advantages of simplified procedure, in situ repair and no secondary pollution, attracts the attention of widespread researchers [31]. In essence, direct regeneration includes the steps of separating spent battery components through meticulous dismantling, screening out high-value cathode materials, replenishing lost metal elements through solid-state sintering, hydrothermal method and other technologies, restoring the material structure, and obtaining battery materials with good performance. Pyrometallurgical and hydrometallurgical methods mainly focus on the “extraction” of valuable metal elements from battery materials, which are then processed into precursors to obtain the battery materials. Direct regeneration eliminates the step of metal ion extraction and focuses on the “repair” of battery material [32]. Under the condition of preserving the original material structure, the structural defects can be repaired according to the specific conditions of different materials, and the repaired cathode materials can be obtained directly [33]. Less chemical reagents are used in the regeneration process, which effectively reduces the secondary pollution to the environment. Meanwhile, the reaction temperature is significantly lower than that of pyrometallurgy, resulting to much less energy consumption. The whole regeneration procedure is short, and only requires precise separation and relithiation to realize the regeneration of decommissioned materials. Since direct regeneration utilizes spent battery materials to directly regenerate the cathode materials, it truly realizes the closed-loop recycling of spent LIBs [34–37]. As depicted in Fig. 1b, the three recycling methods are compared in terms of cost, energy consumption, water consumption, greenhouse gas emissions (GHG) and sulfur oxide emissions, clearly exhibiting the advantages of direct regeneration method [30].

In this paper, we introduce three representative cathodes, namely LiFePO_4_ (LFP), LiNi_*x*_Co_*y*_Mn_*z*_O_2_ (NCM), and LiCoO_2_ (LCO), and analyze the potential reasons for battery failure in these cathodes. Taking the steps of recycling cathode materials of spent LIBs as a clue, starting from the pretreatment process, the current major pretreatment methods are firstly discussed, and then a comprehensive overview of current research progress of five direct regeneration methods, including solid-state sintering, hydrothermal, eutectic molten salt, electrochemical and chemical lithiation regeneration, is also introduced. As most of the current research is still in the experimental stage, the existing relevant literatures mainly focus on the latest progress of direct regeneration from a perspective of theoretical research, there is still a lack of related studies on the practical implementation of battery recycling. For the first time, we propose to use the practical production line of Tianjin Sai De Mei New Energy Technology Co., Ltd. as a case to demonstrate the application of direct regeneration in the industrial production of recycled batteries. Looking forward to the potential of direct regeneration in large-scale industrial applications in the future.

## Failure Reasons of Battery Cathodes

**Fig. 2:**
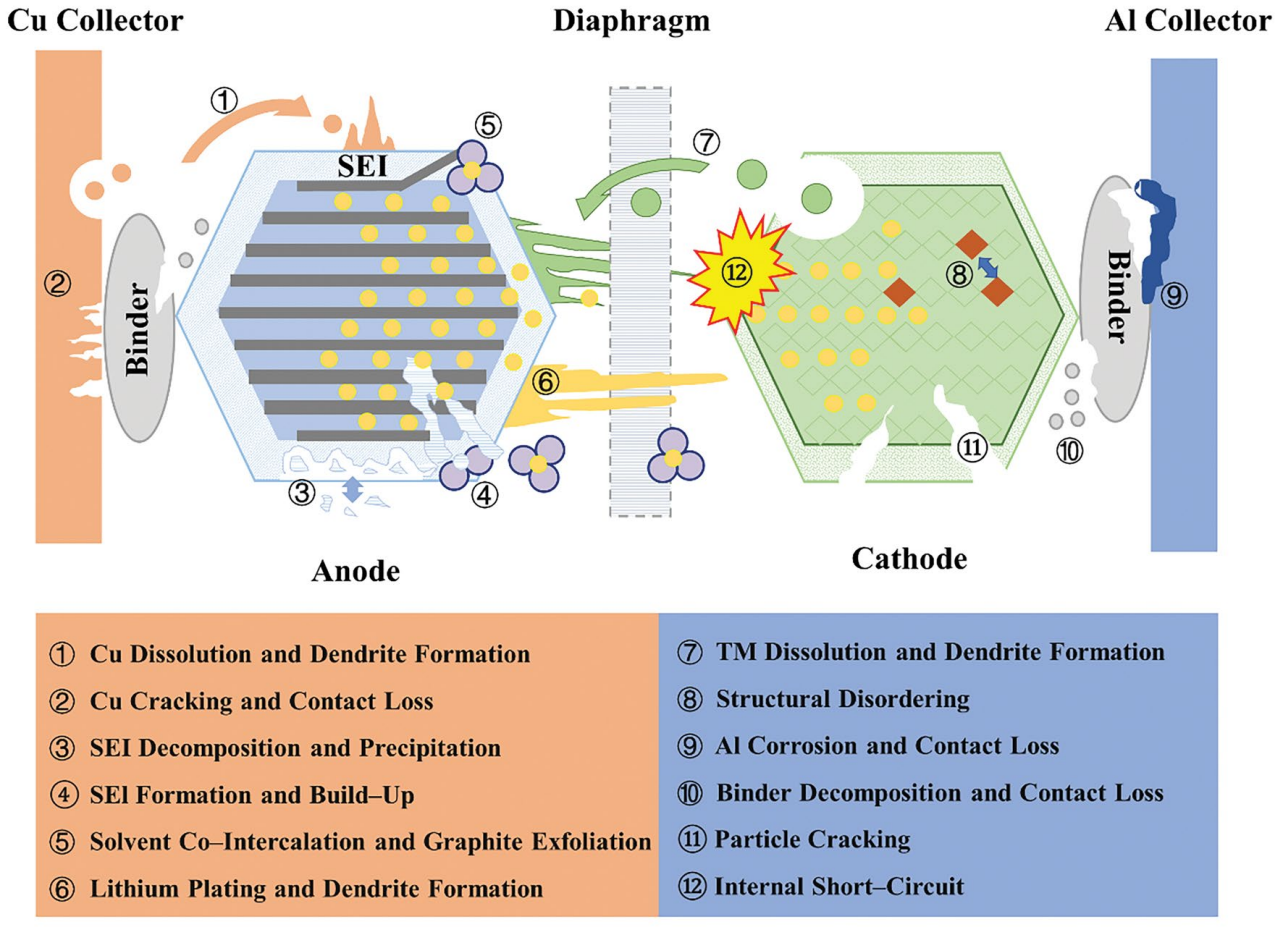
Schematic diagram of battery failure reasons [38]. Copyright 2017, Elsevier

During long-term cycling of LIBs, the repeated insertion and extraction of Li^+^ can lead to irreversible loss of Li^+^. The loss of Li^+^ can cause structural changes in the electrode materials to varying degrees, ultimately affecting the battery performance. The failure of LIBs is typically attributed to cathode failure, anode failure, separator failure and electrolyte failure (Fig. 2) [38]. In the following discussion, we mostly focus on the failure reasons of three representative cathodes: LFP, NCM, and LCO.

### LiFePO_4_

**Fig. 3:**
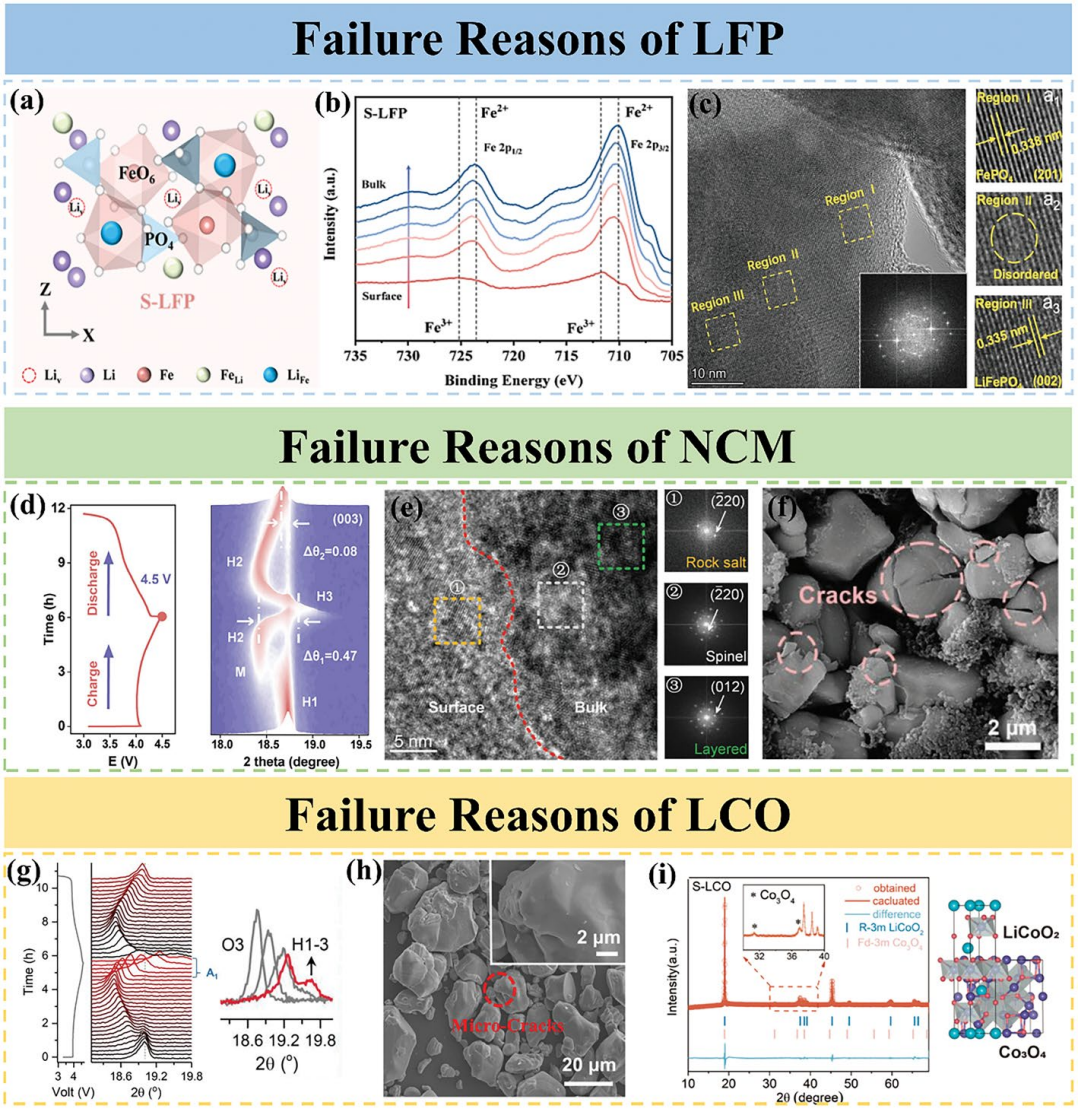
**a** Schematic diagram of lithium vacancy and Li/Fe antisite in LFP [39]. Copyright 2024, Elsevier. **b** In-depth Fe 2*p* XPS spectra of S–LFP (Etch depth: 0–120 nm) [42]. Copyright 2023, Elsevier. **c** HRTEM image of S–LFP and the corresponding FFT pattern. The images (a_1_–a_3_) are the HRTEM images of the specific regions marked with the dashed rectangle [43]. Copyright 2023, WILEY–VCH Verlag. **d** In situ XRD patterns (17.8°–19.7°) with corresponding voltage curves in the voltage range of 3.0 − 4.5 V for NCM during charge/discharge [47]. Copyright 2023, WILEY–VCH Verlag. **e** HRTEM images of S–NCM, the images below are the FFT images of the corresponding areas in the dashed boxes [48]. Copyright 2021, WILEY–VCH Verlag. **f** SEM images for NCM [49]. Copyright 2024, WILEY–VCH Verlag. **g** In situ XRD patterns and the corresponding galvanostatic charge/discharge curves of LCO cathodes during the initial cycling process [50]. Copyright 2024, WILEY–VCH Verlag. **h** Scanning electron microscope images of S–LCO [51]. Copyright 2023, WILEY–VCH Verlag. **i** XRD Rietveld refinement results for S–LCO, with S–LCO crystal structure on the right [52]. Copyright 2023, American Chemical Society

LiFePO_4_ is an olivine-type material, which is one of the three mainstream cathode materials for LIBs. It has been widely used in large-scale applications due to its long cycle life, good thermal stability and high specific capacity. The primary reason for the failure of LFP is the presence of lithium vacancies (Li_V_) and Li/Fe antisite defects. In the crystal structure of LiFePO_4_, Li^+^ occupies the 4a site of the octahedral common side and Fe^2+^ occupies the 4c site of the octahedral common side. Owing to the loss of active lithium source, a Li_v_ establishes at the 4a site (Fig. 3a) [39, 40]. When operating at high current densities, the displaced Li ions are unable to return to their original positions, leading to the accumulation of excess Li_V_ within the LFP structure. This excess Li_V_ promotes the transformation of a portion of Fe^2+^ to Fe^3+^. As a result, the formation of FePO_4_ phase occurs (Fig. 3b) [41, 42]. Since the loss of lithium occurs mainly at the surface or edge region of the particles, the as-formed FePO_4_ phase is wrapped around the outside of LiFePO_4_ phase, and a disordered zone is also formed between the bulk LiFePO_4_ phase and the near surface of FePO_4_ phase (Fig. 3c) [43]. Due to the strong electrostatic repulsion of Fe^3+^, a large amount of activation energy is required for the migration of Fe ions to the initial sites, hindering the reduction of Fe^2+^ to the initial sites during the reduction process. This phenomenon also stimulates the migration of some Fe^2+^ to the unoccupied Li-ion sites, which triggers the generation of Li/Fe antisite defects. During the charging and discharging process, the LFP provides only a one-dimensional transport pathway for Li^+^, and the diffusion rate of Li^+^ is slow [44, 45]. These newly formed Li/Fe antisite defects further impede the Li-ion transport channel, resulting in a decrease in the ion diffusion coefficient of the material. With the increase of cycle numbers, the formation of Li/Fe antisite defects becomes more prominent, which leads to a gradual decline in capacity and a deterioration in cycling stability and rate performance [46]. In the subsequent recovery process, in addition to solving the problem of lithium loss, we also need to pay attention to the reduction of Fe. The presence of a reducing atmosphere is conducive to the reduction of Fe^3+^ to Fe^2+^, transforming FePO_4_ phase into LiFePO_4_ phase as much as possible, facilitating the return of Fe to its original position, inhibiting the generation of Li/Fe antisite defects, and solving the problem of Li^+^ transport channel blockage.

### LiNi_x_Co_y_Mn_z_O_2_

LiNi_x_Co_y_Mn_z_O_2_ cathodes, which are layered lithium-based oxides containing transition metal elements (TM, TM = Ni, Co, Mn), are commonly used due to their higher unit volume, larger specific capacity, and better rate performance compared to LFP. Increasing the Ni content in NCM cathodes generally results in a higher specific capacity [53–55]. Currently, researchers attribute the main causes of failure in NCM cathodes to the element loss and structural changes due to Li^+^/Ni^2+^ mixing. During long-term charging/discharging, Li^+^ needs to undergo repeated insertion and extraction, often requiring a phase transition of H1–M–H2–H3–H2–H1 (Fig. 3d). The formation of the H3 phase is often accompanied by a sharp contraction of the c–axis, which irreversibly affects the material’s structure. Due to the similar atomic radii of Ni^2+^ (0.76 Å) and Li^+^ (0.69 Å), Ni^2+^ migrates from the TM layer to the Li layer through neighboring tetrahedral sites, occupying the Li vacancies formed due to Li loss. This phenomenon, known as Li^+^/Ni^2+^ mixing, destroys the layered phase, resulting in the formation of both a rock salt phase and a spinel phase on the surface (Fig. 3e) [48, 56, 57]. The transformation of the material from the original layered *R–3m* space group to a tightly packed spinel *Fm–3m* space group leads to a shortening of ionic distances and an enhancement of electrostatic interactions, further exacerbating Li^+^/Ni^2+^ mixing [58]. Additionally, the expansion of the rock salt phase inward leads to lattice deformation, stress concentration, and the formation of cracks within the material (Fig. 3f) [49, 59]. These surface degradation effects decrease the material’s capacity, increase electrochemical impedance, and hinder Li^+^ insertion, negatively impacting the material properties [60]. In subsequent regeneration processes, providing a sufficient oxidizing environment is necessary to promote the transformation of Ni^2+^ to Ni^3+^, eliminate the Li^+^/Ni^2+^ mixing phenomenon, and transform the rock salt phase back into the layered phase. Furthermore, repairing the cracks inside the material particles and eliminating the stress concentration phenomenon are essential to restore the material’s original structure and performance.

### LiCoO_2_

LiCoO_2_ (LCO) has been the first-generation cathode material for commercial LIBs and has dominated the cathode material for 3C product batteries for nearly 30 years. Structural collapse and the formation of an unfavorable phase interface are the primary factors that contribute to the deterioration of LiCoO_2_ performance. During the charging process, an increase in the number of Li_v_ causes the CoO_2_ slab to slide and generates strong electrostatic repulsion between the oxygen layers. This results in a phase transition from the O3 phase to the H1–3 phase in LCO (Fig. 3g) [50, 61]. This phase transition induces a gradual increase in the c–axis lattice parameter of Li_*x*_CoO_2_ until *x* = 0.5, followed by a rapid contraction after *x* = 0.35. Consequently, a significant accumulation of stresses occurs, leading to material cracking and structure collapse (Fig. 3h) [51, 62, 63]. Due to the overlap of the O_2_ 2*p* and Co^3+^/Co^4+^ 3*d* electronic bands, high pressures triggers the oxidation of lattice oxygen and the formation of oxygen radicals. This process leads to the escape of oxygen from the surface lattice of LCO and the subsequent formation of the Co_3_O_4_ spinel phase (Fig. 3i) [52, 64–66]. During prolonged cycling, as the Co valence state changes, the surface structure of LCO undergoes a transformation from a layered to a spinel and rock salt phase structure. The Co^2+^ resulting from reduction readily depart from the material’s surface, entering the electrolyte and migrating toward the negative electrode. This process causes the destruction of the solid electrolyte interface (SEI) film and leads to the eventual deposition on the surface of the negative electrode. These factors further accelerate the degradation of electrochemical performance [67, 68]. The regeneration process requires careful attention to elemental replenishment, the release of residual stresses, and the repair of material cracks and structural defects.

## Pretreatment

In consideration of various components present in a battery, it is crucial to perform pretreatments before initiating a battery recycling process. The pretreatment procedure typically consists of three steps: discharge, disassembly and stripping. According to the surveys, retired batteries usually retain 70%–80% of their initial capacity. However, when it comes to batteries obtained from portable electronics, the pretreatment process can be bypassed due to their small size and relatively limited retained capacity. These batteries can be directly broken down for subsequent recycling. On the other hand, the packed power batteries obtained from electric vehicles or power stations require careful handling. If not fully discharged, the subsequent disassembly and exfoliation process may result in collisions, punctures or even short circuits due to their high voltage and capacity. As a result, thermal runaway and the release of toxic or corrosive substances will occur, which further leads to fire and explosion [69]. From a safety perspective, it is crucial to perform a battery discharge prior to the electrode exfoliation [70, 71].

Currently, most researchers use conductive aqueous solutions for battery discharge. When the battery is immersed into a conductive aqueous solution, a short circuit occurs between the cathode and anode, with the purpose of releasing the remaining power within the batteries. Sodium chloride (NaCl) is considered as an inexpensive and valuable electrolyte for the production of conductive solutions on a large scale. It has been demonstrated that the discharge time can be reduced by increasing the concentration of NaCl solution [72]. However, a high concentration of Cl^–^ can result in substantial electrode corrosion, which has been proven to be detrimental [73]. In addition to conductive salt solutions, both acidic and alkaline solutions can also serve as effective discharge media. Wu et al. [74] discovered that sodium hydroxide (NaOH) exclusively caused serious corrosion on Al sheets, while it had no corrosive impact on Fe sheets. Based on this distinctive characteristic, the NaOH + Na_2_SiO_3_ composite system was devised. The Al^3+^ combine with silicates to generate an aluminosilicate gel, which forms a protective layer on the surface of Al sheets. This layer effectively inhibits further corrosion of batteries during the discharge process. In addition to conductive aqueous solutions, conductive solid powders (such as copper and graphite), can also be utilized to facilitate the discharge process. Graphite, as a highly conductive non-metallic conductor, can be mixed with spent LIBs to establish a connection between the cathode and anode, thereby facilitating the battery discharge. However, it is important to note that the discharge process facilitated by graphite is typically rapid, leading to a sudden generation of substantial heat. As a result, this rapid increase of temperature poses an increased risk of explosion. Therefore, the graphite-assisted discharge method is exclusively suitable for small-size batteries [16].

**Fig. 4:**
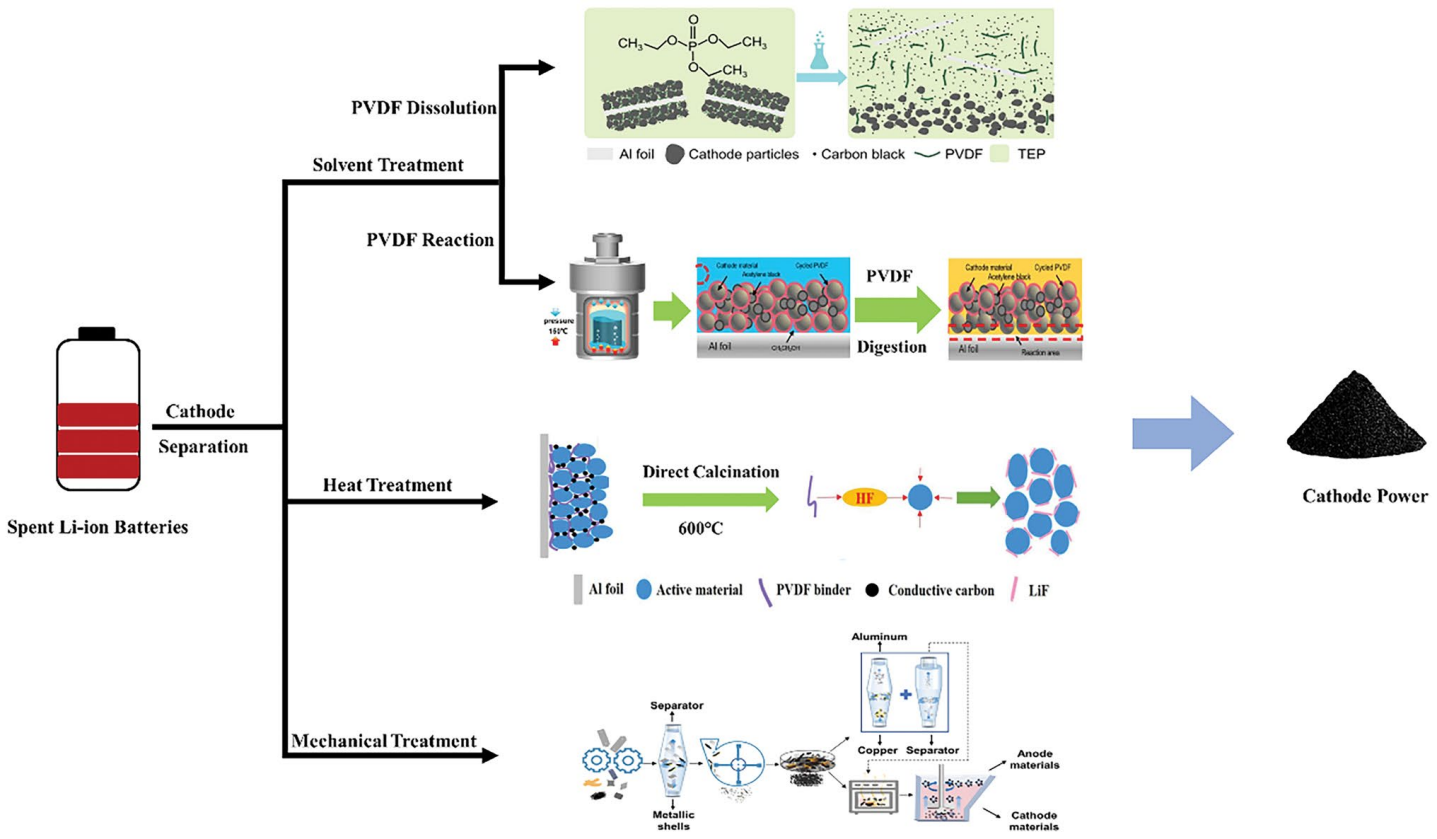
Schematic diagram of representative exfoliation methods for cathode plates [75–78]. Copyright 2021, American Chemical Society. Copyright 2022, RSC publishing. Copyright 2016, American Chemical Society. Copyright 2021, Elsevier

After the battery is discharged, the disassembly process is usually carried out manually, where the battery shell, cathode plate, anode plate, separator and other accessories are separated. Then, the active material is exfoliated from the current collector. In this context, our main focus is on the exfoliation of cathode material from the Al foil. To enhance the adhesion between the cathode material and Al foil during the production of cathode plate, the inclusion of a binder, such as PVDF, appears to be essential. Consequently, it becomes challenging to directly separate the cathode material from Al foil, and additional measures need to be taken to facilitate the exfoliation of the cathode material from Al foil. In the following content, several representative exfoliation methods are introduced, as shown in Fig. 4.

### Solvent Treatment

#### PVDF Dissolution

Based on “like dissolves like” principle, highly polar solvents such as N-methyl-pyrrolidone (NMP) [79], N, N-dimethylformamide (DMF) [73], dimethyl sulfoxide (DMSO) [74] and dimethyl acetamide (DMAC) [80] were utilized to dissolve the PVDF and facilitate the separation of the cathode material from the Al foil. Among them, NMP shows the most effective dissolution effect on PVDF. However, it has been observed that the active material treated by NMP solvent often exists in the form of fine power, which is susceptible to causing clogging during filtration and greatly enhances the challenges associated with cathode recycling. In addition, NMP solvent is relatively expensive and has a high viscosity. The dissolution by NMP often requires a large amount of consumption, and the recycle of NMP is proven to be very challenging, which greatly hinders its widespread application. Furthermore, the toxicity of NMP poses a risk to human health [75]. Therefore, more efficient and environmentally friendly solvents are needed to replace NMP.

Among numerous candidates, triethyl phosphate (TEP) has gathered great attentions due to its comparable properties with NMP. Bai et al. [75] utilized TEP as a solvent to dissolve PVDF. The resulting active material was then separated from Al foil through filtration and centrifugation. The recovery process demonstrated no leaching of metals, and no observable morphology or structure change was detected in the active material. More importantly, TEP can be recycled after a simple treatment, making it sustainable and environmentally friendly. In addition to TEP, dimethyl isosorbide (DMI) with an inexpensive price, has similar Hildebrand solubility parameters to PVDF, and is considered as a cost‒effective solvent [81]. Buken et al. [82] discovered that DMI has the ability to permeate into the crystalline region of PVDF. The formation of hydrogen bonds between DMI and PVDF strengthens the interactions between the solvent and polymer, thereby reducing the interactions between polymer chains. This phenomenon facilitates the detachment of PVDF from the Al current collector. Due to its nontoxicity and production from sugar, DMI is often considered as a green alternative to NMP.

#### PVDF Reaction

Due to the fluorine atom in PVDF, the hydrogen atom in the CH_2_ group of PVDF becomes positively charged, rendering it susceptible to OH^–^. This interaction triggers a reaction between the OH^–^ atom and the hydrogen atom, resulting in the destruction of the bonding configuration of PVDF and leading to its structural decomposition. Consequently, the material separates from the Al foil.

Ethanol is considered as an environmentally friendly solvent which contains –OH. In a study conducted by Qin et al. [76], they discovered that when PVDF was exposed to high pressure at 150 °C, it undergoes an elimination reaction with the –OH in ethanol, resulting in the replacement of F atom on the unsaturated double bond with –OH. Due to the inherent instability of –OH group on the unsaturated double bond, it will be further oxidized into a ketone structure. This oxidation process breaks down the PVDF structure, ultimately resulting in a complete material stripping rate of 100%. LiOH is a typical salt that contains OH^–^. Ji et al. [83] employed a molten salt consisting of LiOH and LiNO_3_, which had a melting point of 175 °C. The OH^–^ generated from this molten salt promoted the conversion of PVDF into alkylamine groups at 260 °C, thereby effectively separating the cathode material from Al foil.

In addition to the use of OH^–^, the hydroxyl radical (·OH) also plays a vital role in facilitating the rapid degradation of PVDF. This degradation process can bring about in situ detachment of cathode material from Al foil. Chen et al. [84] employed Fe^2+^ as a Fenton reagent to react with H_2_O_2_, which generates highly reactive ·OH. The effectiveness of this reaction was further amplified through the application of ultrasonic waves, which promotes the decomposition of H_2_O_2_ and production of ·OH. These generated ·OH effectively reacted with PVDF, enabling the selective removal of PVDF. An impressive stripping rate of 97% can be achieved on both Al foil and cathode material, and the collected cathode material demonstrates a high purity and is not vulnerable to contamination from wastewater.

### Heat Treatment

Under high-temperature heat treatment, PVDF will be decomposed into small organic molecules. This decomposition process leads to a decrease of bonding forces between the active material and PVDF binder, which facilitates the separation of the active material from Al foil [85].

Previous studies have shown that the decomposition of PVDF begins at about 350 °C, and a complete breakdown of PVDF occurs at about 600 °C. Hu et al. [86] observed the fragmentation of the long-chain structure of PVDF at 450 °C, resulting in the formation of HF gas, CF_2_=CF_2_ molecule, fluorobenzene compounds and some short-chain alkanes. Additionally, the recovery rate of Al foil and active material reached 100%. Zhang et al. [77] conducted a comparison of three pretreatment methods, namely NMP solvent dissolution, NaOH solution treatment and heat treatment. They found that the recycled cathode material obtained through heat treatment at 600 °C exhibited the most favorable electrochemical performance. It should be noticed that PVDF has the potential to be decomposed into corrosive HF gas when exposed to high temperatures, which not only has a risk of equipment corrosion, but poses a threat to the surrounding environment. To address this issue, Wang et al. [87] put forward the use of CaO as a reaction medium. This medium has the ability to absorb the volatilized HF gas generated during heat treatment process.

### Mechanical Treatment

Mechanical treatment belongs to a physical method that utilizes mechanical force to separate the active material from Al foil. The active material, in the form of granules, can be collected through sieving or other appropriate procedures [88].

Crushing is widely recognized as a conventional mechanical treatment technique. Zhu et al. [78] utilized a hammer mill to pulverize battery electrode plates into particles with an average size of less than 2 mm. Subsequently, these particles were sieved by a screen with a size of 0.25 mm. The collected powder with a size smaller than 0.25 mm, primarily consisted of cathode and anode active materials. The variable diameter pneumatic classification method is used to further separate the substances with different densities. Heat treatment is implemented on the active material to eliminate the adhered binder. Through the utilization of foaming agents for flotation, taking into account the disparity in hydrophilicity/hydrophobicity of the material surface, the recovery rate of the active substance can surpass 92%. Grinding, an alternative mechanical treatment technique, can be also considered. The rapid temperature fluctuations cause modification of material properties. Furthermore, low-temperature treatment promotes the transition of PVDF from a highly elastic configuration to a vitreous state, thereby reducing its adhesive characteristics. Wang et al. [89] proposed a low-temperature grinding method to eliminate the active material. In this approach, the Al foil remained unchanged, while the cathode material was stripped away in a form of powder. The conventional high-temperature grinding method yields a stripping rate of 25%. However, the low-temperature grinding method remarkably elevates this stripping rate to 87%. In present studies, scientists have harnessed the ultrasound to stimulate chemical reactions and mechanical interactions at the interface between solids and liquids, which accelerates the separation of active material from Al foil. Lei et al. [90] employed high-power ultrasound to achieve rapid delamination of the active material from Al foil. This method takes advantages of the cavitation phenomenon, which quickly and selectively disrupts the adhesive at the electrode interface. Consequently, the separation of electrodes can be achieved within seconds, resulting in a substantial reduction of material processing duration and a notable increase of material stripping rate.

The effectiveness of the direct regeneration process largely depends on the precise disassembly at the front end. However, during the pretreatment process, a certain amount of impurity Al may remain in the material to varying degrees. Current research has indicated that small amounts of impurity Al can actually have a positive impact on the direct material regeneration. Xing et al. [91] discovered that 3 wt.% Al in the material can inhibit the mixing of Li^+^/Ni^2+^, fill the vacancies in the TM layer, widen the layer spacing, promote ionic diffusion, stabilize the layered structure, and improve the cycling stability of the material. On the contrary, excessive Al can lead to the formation of an Al(OH)_3_ impurity phase on the surface of NCM particles, resulting in the deterioration of the laminar structure and increased Li/Ni mixing. Therefore, it is crucial to improve the separation between the cathode material and the Al foil, reduce the amount of impurity Al in the cathode material, and explore the threshold for impurity presence in order to minimize the impact of impurities.

## Direct Regeneration Methods

After the aforementioned pretreatments, spent LIBs can successfully realize the segregation of active material from Al foil, thus effectively avoiding the introduction of impurity components throughout the recycling procedure. The collected cathode powder possesses high purity, which is crucial for subsequent direct regeneration. In recent times, direct regeneration approaches have garnered significant interests due to the *in situ* regeneration feature, short process and less pollution. They mainly include solid‒state sintering, hydrothermal, eutectic molten salt, electrochemical and chemical lithiation methods. This section will elaborate on these five specific direct regeneration methodologies.

### Solid‒State Sintering Method

Solid‒state sintering achieves the revitalization of spent materials through a simple solid-phase reaction. Firstly, it is imperative to precisely quantify the amount of lost lithium in the materials to be repaired. By stoichiometric calculation, the supplementary quantity of lithium can be determined, and the lithium salts are added in a specific ratio. Subsequently, during high-temperature sintering process, the solid lithium source melts with the material particles, which facilitates the diffusion of Li^+^ into the vacancies within the material structure. This process effectively replenishes the lost lithium and restores the damaged structure, ultimately reinstating the material performance [92–94].

**Fig. 5:**
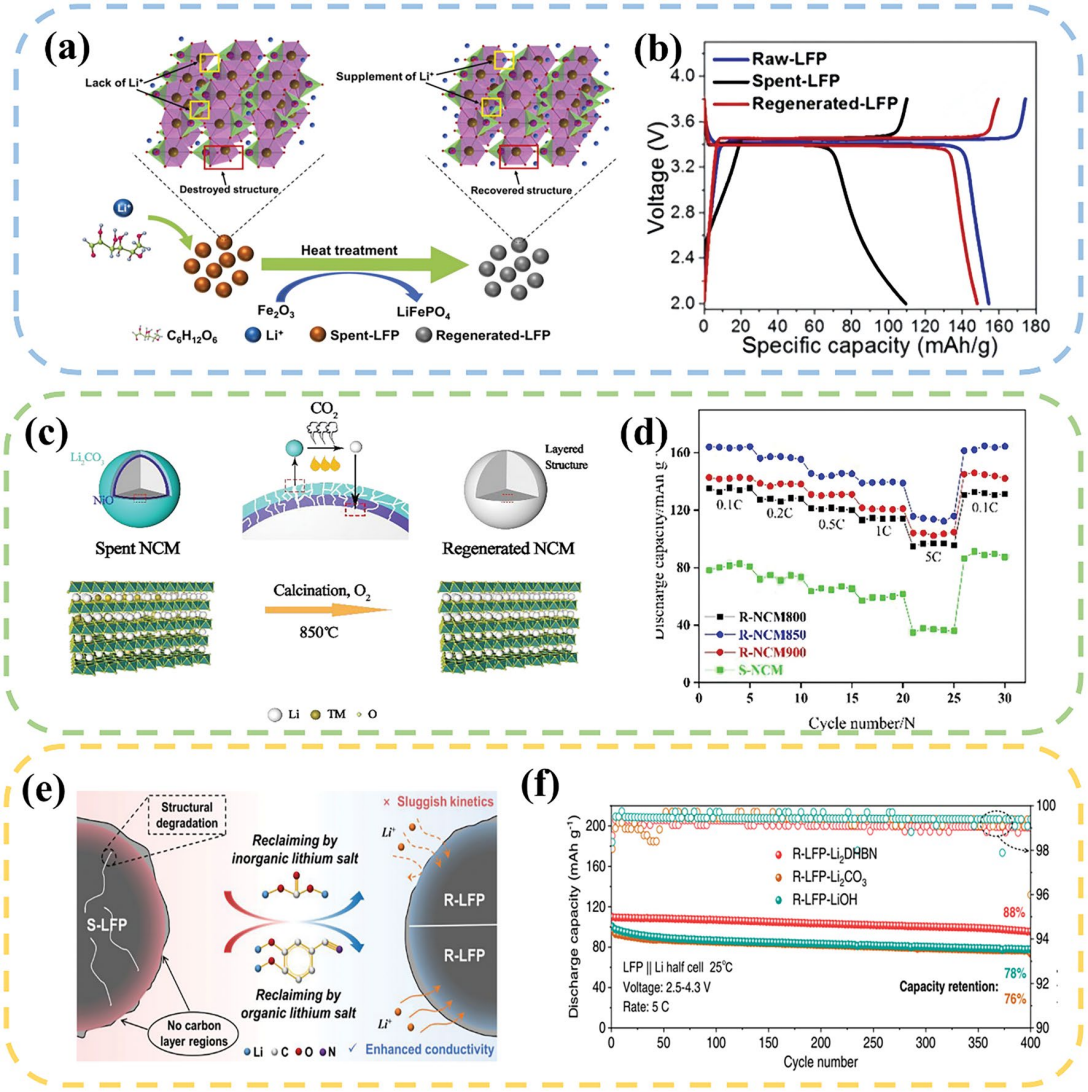
**a** Schematic diagram of the regeneration of spent LFP by adding common lithium salt. **b** Charge/discharge curves of Raw-LFP, Spent-FP and Regeneration-LFP at 0.05 C [96]. Copyright 2022, Elsevier. **c** Schematic diagram of the direct regeneration of NCM111 by surface lithium residue. **d** Rate performance of S–NCM, R–NCM800, R–NCM850, and R–NCM900 samples [98]. Copyright 2021, RSC Publishing. **e** Schematic of the regeneration mechanism of S–LFP by using inorganic and organic lithium salts. **f** Cycling performance of different electrodes at 5 C [99]. Copyright 2023, Springer Nature

The supplementation of lithium is typically completed by introducing an external lithium source. Basically, the utilization of LiNO_3_ leads to the generation of harmful gases, and the use of LiOH is hindered by its corrosive nature and relatively high cost. Li_2_CO_3_ is widely employed in solid-state sintering to compensate the lost lithium. Tang et al. [95] employed jet milling and ball milling techniques to decrease the particle size of spent NCM523 cathode, which was then sintered at 920 °C for 12 h. This process helps replenish the lost lithium and restore the layered structure of NCM523. The regenerated particles exhibited a distinct single crystal morphology and ensured a well–dispersed arrangement of native particles. The electrochemical tests demonstrated that the initial discharge specific capacity reached 155 mAh g^−1^, and the capacity retention rate after 100 cycles was 90%. Qi et al. [96] used glucose as a reducing agent and mixed it with spent LFP, then calcined it at 900 °C for 6 h, during which Fe_2_O_3_ was converted to LiFePO_4_ (Fig. 5a). The regenerated LFP had a discharge capacity of 148 mAh g^−1^ at 0.05 C, which was 96% of the initial capacity of pristine LFP (Fig. 5b). Kong et al. [97] sintered spent LCO with a 50% loss of lithium at 850 °C for 8 h. After this sintering process, the Co_3_O_4_ impurity disappeared, and the uniformly distributed LiCoO_2_ particles with a smaller size and clearer layered established. It was found that the regenerated LCO cathode showed superior electrochemical performance as compared to commercial LCO.

Some researchers also utilize the residual lithium compounds on the surface of retired materials as a lithium source for the regeneration process. Chi et al. [98] used the residual Li_2_CO_3_ coating on the material surface as the lithium source to regenerate NCM111 at 850 °C for a duration of 12 h, which eliminates the addition of extra lithium salt. The decomposition of Li_2_CO_3_ replenishes the lithium defects in the regenerated material, and induces the transformation of NiO from rock–salt phase to ordered layer structure, thus significantly enhancing the electrochemical performance (Fig. 5c, d). Traditional lithium salts only serve the single role of replenishing lost lithium elements. However, when it comes to LFP, additional reducing agents or carbon sources are often required. Ji et al. [99] developed a multifunctional organic lithium salt, known as 3,4–dihydroxybenzonitrile dilithium, which directly regenerated the spent LFP at 800 °C under an Ar/H_2_ atmosphere. During the increase of sintering temperature, the lithium ions in the salt fill the vacancies, and cyano groups generates a reducing atmosphere, inhibiting the formation of Fe (III) phase (Fig. 5e). Meanwhile, the amorphous conductive carbon derived from the salt encapsulates the surface of LFP particles, which enhances the diffusion of Li ions and accelerates the transfer of electrons. As a result, the regenerated LFP cathode exhibits excellent cycling stability, with a capacity retention rate of 88% after 400 cycles at a current density of 5 C (Fig. 5f). Clearly, the use of lithium-containing impurities on the material surface or multifunctional organic lithium salts offers more lithium sources for the regeneration process. Table 1 summarizes the experimental conditions and regeneration effects of current solid-state sintering method.

**Table 1:** A summary of solid-state sintering method

Type	Parameters	Performance	References
LFP	Reagent: 3,4–dihydroxybenzonitrile dilithium 800 °C, 6 h, Ar/H_2_	Initial discharge capacity: 157 mAh g^−1^; Current density: 5 C; Cycle number: 400; Capacity retention rate: 88%	[99]
LFP	Reagent: Li_2_CO_3_, CNTs, glucose 350 °C, 2 h; 650 °C, 12 h	Initial discharge capacity: 155 mAh g^−1^; Current density: 0.5 C; Cycle number: 300; Capacity retention rate: 88%	[100]
LFP	Reagent: Li_2_CO_3_ 650 °C, 1 h	Initial discharge capacity: 147 mAh g^−1^; Current density: 0.2 C; Cycle number: 100; Capacity retention rate: 95%	[101]
LFP	Reagent: Li_2_CO_3_ 700 °C, 3 h	Initial discharge capacity: 124 mAh g^−1^; Current density: 0.5 C; Cycle number: 2000; Capacity retention rate: 83%	[102]
LFP	Reagent: Li_2_CO_3_ 350 °C, 5 h; 650 °C, 10 h	Initial discharge capacity: 96 mAh g^−1^; Current density: 10 C; Cycle number: 2000; Capacity retention rate: 80%	[103]
LCO	Reagent: Li_2_CO_3_ 850 °C, 8 h	Initial discharge capacity: 139 mAh g^−1^; Current density: 1 C; Cycle number: 100; Capacity retention rate: 90%	[97]
LCO	Reagent: Li_2_CO_3_ 850 °C, 12 h	Initial discharge capacity: 150 mAh g^−1^; Current density: 0.1 C; Cycle number: 100; Capacity retention rate: 93%	[104]
LCO	Reagent: Li_2_CO_3_ 850 °C, 12 h	Initial discharge capacity: 160 mAh g^−1^; Current density: 0.2 C; Cycle number: 50; Capacity retention rate: 91%	[105]
LCO	Reagent: Li_2_CO_3_ 900 °C, 6 h	Initial discharge capacity: 140 mAh g^−1^; Current density: 0.1 C; Cycle number: 100; Capacity retention rate: 97%	[106]
LCO	Reagent: Li_2_CO_3_ 900 °C, 12 h	Initial discharge capacity: 152 mAh g^−1^; Cycle number: 80; Capacity retention rate: 98%	[107]
LCO	Reagent: Li_2_CO_3_, MgO, TiO_2_ 1000 °C, 10 h	Initial discharge capacity: 178 mAh g^−1^; Current density: 1 C; Cycle number: 100; Capacity retention rate: 96%	[108]
LCO	Reagent: Li_2_CO_3_, MgO 900 °C, 12 h	Initial discharge capacity: 203 mAh g^−1^; Current density: 1 C; Cycle number: 100; Capacity retention rate: 97%	[35]
LCO	Reagent: Li_2_CO_3_, MgO, Al_2_O_3_ 900 °C, 10 h	Initial discharge capacity: 220 mAh g^−1^; Current density: 0.5 C; Cycle number: 100; Capacity retention rate: 94%	[63]
NCM	Reagent: Li_2_CO_3_ 950 °C	Initial discharge capacity: 140 mAh g^−1^; Current density: 0.2 C; Cycle number: 50; Capacity retention rate: 97%	[109]
NCM	Reagent: Li_2_CO_3_ 950 °C, 10 h	Initial discharge capacity: 169 mAh g^−1^; Current density: 1 C; Cycle number: 200; Capacity retention rate: 80%	[110]
NCM	850 °C, 12 h, O_2_	Initial discharge capacity: 170 mAh g^−1^; Current density: 0.5 C; Cycle number: 200; Capacity retention rate: 87%	[98]
NCM	Reagent: Li_2_CO_3_ 920 °C, 12 h	Initial discharge capacity: 155 mAh g^−1^; Current density: 0.5 C; Cycle number: 100; Capacity retention rate: 90%	[95]
NCM	Reagent: Li_2_CO_3_ 800 °C, 10 h	Initial discharge capacity: 165 mAh g^−1^; Current density: 0.2 C; Cycle number: 100; Capacity retention rate: 81%	[111]
NCM	Reagent: LiOH 850 °C, 5 h	Initial discharge capacity: 154 mAh g^−1^; Current density: 1 C; Cycle number: 100; Capacity retention rate: 95%	[112]
NCM	850 °C, 6 h	Initial discharge capacity: 170 mAh g^−1^; Current density: 0.1 C; Cycle number: 1000; Capacity retention rate: 93%	[113]
NCM	Reagent: LiOH 800 °C, 8 h	Initial discharge capacity: 147 mAh g^−1^; Current density: 0.2 C; Cycle number: 100; Capacity retention rate: 95%	[114]

### Hydrothermal Method

During hydrothermal regeneration process, the spent cathode materials are mixed with a lithium-containing solution under liquid-phase condition. The Li^+^ in the solution can evenly diffuse into the Li vacancies within the cathode material. Additionally, the high-pressure environment of hydrothermal reaction reduces the activation energy barrier for Li^+^ diffusion, further promoting the Li^+^ diffusion and compensating the lost lithium. In general, the hydrothermal regeneration can be divided into three types: conventional hydrothermal regeneration, low-temperature hydrothermal regeneration and microwave hydrothermal regeneration [115].

**Fig. 6:**
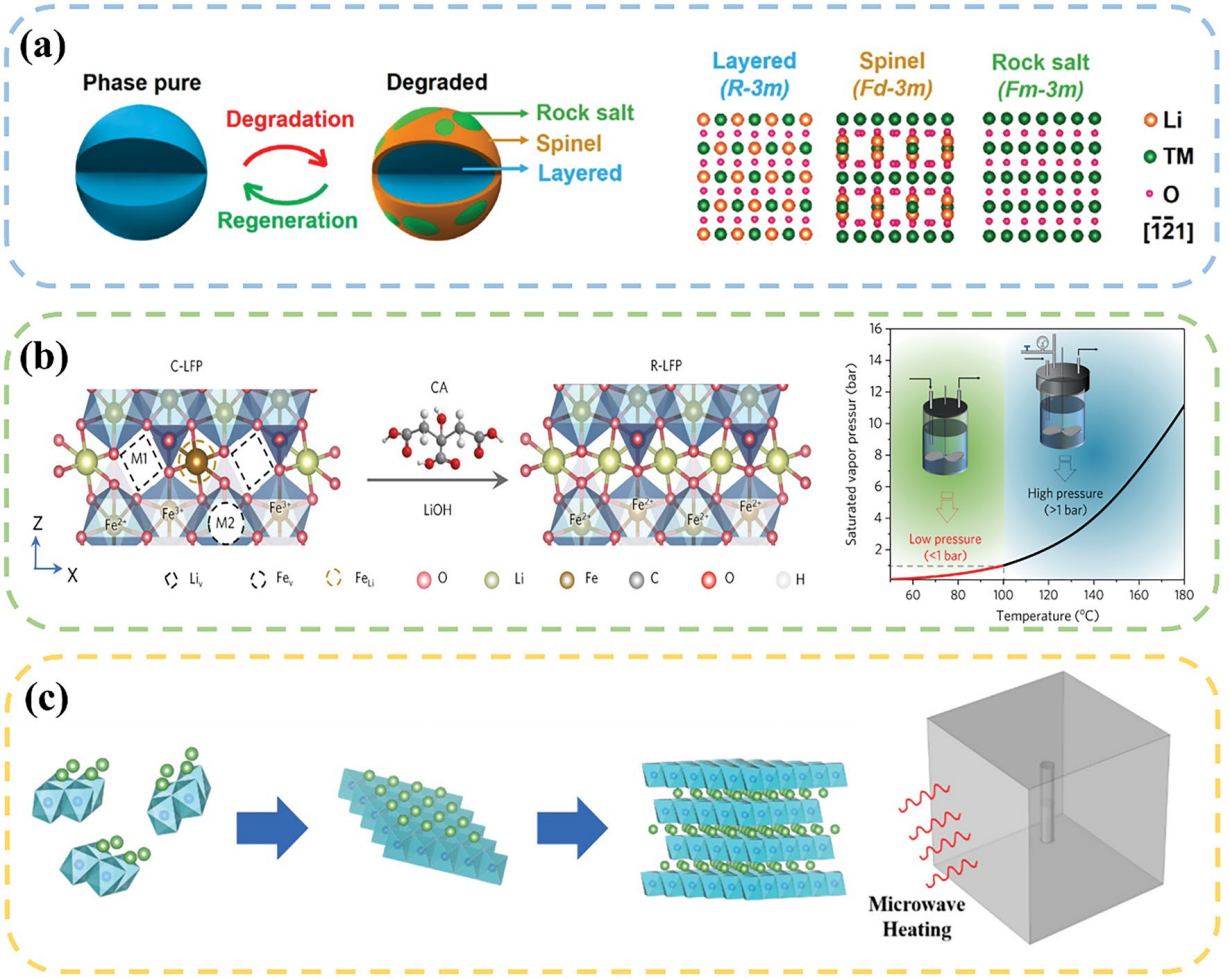
**a** Schematic diagram of the crystal structure change of NCM523 before and after regeneration. The right scheme shows the atomic arrangement of layered, spinel, and rock salt phases along the [1̅2̅1] zone axis [117]. Copyright 2018, American Chemical Society. **b** Schematic diagram of repairing the retired LFP by low-temperature hydrothermal method [46]. Copyright 2020, Elsevier. **c** Schematic diagram of the regeneration of LCO by a microwave hydrothermal method [120]. Copyright 2022, Elsevier

Shi et al. [116] repaired the damaged structure of spent LCO by a hydrothermal reaction at 220 °C for 4 h using LiOH as a lithium source. The electrochemical result demonstrated a decrease of charge transfer resistance and an increase of Li^+^ diffusion rate in the regenerated sample. Likewise, Shi et al. [117] employed an identical hydrothermal condition to regenerate the NCM523 cathode. It was evidenced that the spinel and rock–salt impurities were successfully converted into the layered phase, thereby mitigating the intermixing of Li/Ni ions (Fig. 6a). Xu et al. [118] implemented a cost-reduction strategy by substituting conventional LiOH solution with LiOH/KOH blend solution. The scale-up experiment was also conducted to demonstrate the comparable capacity of the regenerated material to the original level, thereby offering a promising avenue to the large-scale industrial implementation of hydrothermal regeneration technique. Additionally, the control of hydrothermal conditions greatly determines the replenishment of Li^+^ and improvement of electrochemical performance. Jing et al. [119] used Li_2_SO_4_ and hydrazine hydrate as the lithium source and reducing agent to study the optimal conditions for the LFP regeneration. Under the condition of reaction at 200 °C for 3 h (Li source: 12 g L^−1^; reductant: 1.0 mL; S–LFP powder: 5 g; L/S = 6 mL g^−1^), the regenerated LFP exhibited excellent performance, with a discharge capacity of 142 mAh g^−1^ at 1 C and a capacity retention rate of 99% after 200 cycles.

The hydrothermal reaction typically occurs within the temperature range between 180 and 220 °C. A high-pressure reactor capable of withstanding the pressure exceeding 11 atmospheres (equivalent to the saturation pressure of water) is essential for promoting an efficient hydrothermal reaction. Xu et al. [46] proposed a method for the low-temperature hydrothermal regeneration of LFP. LiOH was used as the lithium source and citric acid (CA) was employed as a reducing agent for the recovery process. The CA provides electrons to facilitate the reduction of Fe^3+^ to Fe^2+^, which minimizes the electrostatic repulsion caused by Fe^3+^ and lowers the migration barrier, further promoting the migration of Fe^2+^ from the M1 site to M2 site and enhancing the diffusion of Li^+^. When the reaction temperature decreased to 80 °C, the corresponding reaction pressure fell to below 1 bar. Combined with annealing for a short time, the impurity phase and Li/Fe antisite defects in the regenerated LFP disappeared, resulting in superior electrochemical performance as compared to commercial LFP (Fig. 6b). Liu et al. [120] developed a microwave hydrothermal method for regenerating the spent LCO. This method utilized the microwave heat caused by dipole rotation to align the molecules in the electromagnetic field. Compared to conventional hydrothermal method, the regenerated LCO material obtained by this method exhibits uniform distribution state, well-organized layer-by-layer particle assembly, improved crystallinity and enhanced cycling stability (Fig. 6c). Table 2 summarizes the experimental conditions and regeneration effects of current hydrothermal method.

**Table 2:** A summary of hydrothermal method

Type	Parameters	Performance	References
LFP	Reagent: CH_3_COOLi, ethanol Hydrothermal: 180 °C, 5 h	Initial discharge capacity: 139 mAh g^−1^; Current density: 1 C; Cycle number: 1000; Capacity retention rate: 77%	[44]
LFP	Reagent: Li_2_SO_4_·H_2_O, N_2_H_4_·H_2_O Hydrothermal: 220 °C, 3 h	Initial discharge capacity: 142 mAh g^−1^; Current density: 1 C; Cycle number: 200; Capacity retention rate: 99%	[119]
LFP	Reagent: LiOH, CA Hydrothermal: 70 °C, 10 h	Initial discharge capacity: 159 mAh g^−1^; Current density: 0.5 C; Cycle number: 100; Capacity retention rate: 99%	[46]
LFP	Reagent: CH_3_COOLi, PVP Hydrothermal: 180 °C, 5 h	Initial discharge capacity: 139 mAh g^−1^; Current density: 1 C; Cycle number: 1000; Capacity retention rate: 80%	[44]
LCO	Reagent: LiOH, Li_2_SO_4_ Hydrothermal: 220 °C, 4 h	Initial discharge capacity: 149 mAh g^−1^; Current density: 1 C; Cycle number: 100; Capacity retention rate: 91%	[116]
LCO	Reagent: LiOH Hydrothermal: 220 °C, 45 min	Initial discharge capacity: 142 mAh g^−1^; Current density: 5 C; Cycle number: 100; Capacity retention rate: 95%	[120]
LCO	Reagent: LiOH Hydrothermal: 220 °C, 4 h	Initial discharge capacity: 166 mAh g^−1^; Current density: 1 C; Cycle number: 100; Capacity retention rate: 93%	[121]
LCO	Reagent: LiOH/KOH, nickel, manganese acetates Hydrothermal: 190 °C, 12 h	Initial discharge capacity: 160 mAh g^−1^; Current density: 1 C; Cycle number: 100; Capacity retention rate: 91%	[122]
NCM	Reagent: LiOH Hydrothermal: 100 °C, 8 h	Initial discharge capacity: 154 mAh g^−1^; Current density: 1 C; Cycle number: 200; Capacity retention rate: 83%	[123]
NCM	Reagent: LiOH Hydrothermal: 220 °C, 4 h	Initial discharge capacity: 155 mAh g^−1^; Current density: 1/3 C; Cycle number: 100; Capacity retention rate: 95%	[124]
NCM	Reagent: LiOH Hydrothermal: 220 °C, 3 h	Initial discharge capacity: 166 mAh g^−1^; Current density: 1 C; Cycle number: 500; Capacity retention rate: 91%	[125]
NCM	Reagent: LiOH, Li_2_SO_4_ Hydrothermal: 220 °C, 4 h	Initial discharge capacity: 145 mAh g^−1^; Current density: 1 C; Cycle number: 100; Capacity retention rate: 85%	[117]
NCM	Reagent: LiOH, KOH Hydrothermal: 220 °C, 2 h	Initial discharge capacity: 156 mAh g^−1^; Current density: 1/3 C; Cycle number: 50; Capacity retention rate: 93%	[118]

### Eutectic Molten Salt Method

During the regeneration by eutectic molten salts, lithium-containing molten salts are used as a source of lithium. Due to the formation of a new phase when the eutectic molten salts are mixed, this new phase has a lower melting point compared to a single phase, effectively reducing the reaction temperature during the regeneration process. For instance, when LiNO_3_ and LiOH are combined in a molar ratio of 3:2, the resulting phase exhibits a melting point of 175 °C, which is considerably lower than the melting points of the individual substances (LiNO_3_:264 °C; LiOH:462 °C) [126]. Once the reaction temperature reaches the eutectic point, the molten salt undergoes a transition from a solid state to a molten state, thus facilitating enhanced Li^+^ diffusion.

**Fig. 7:**
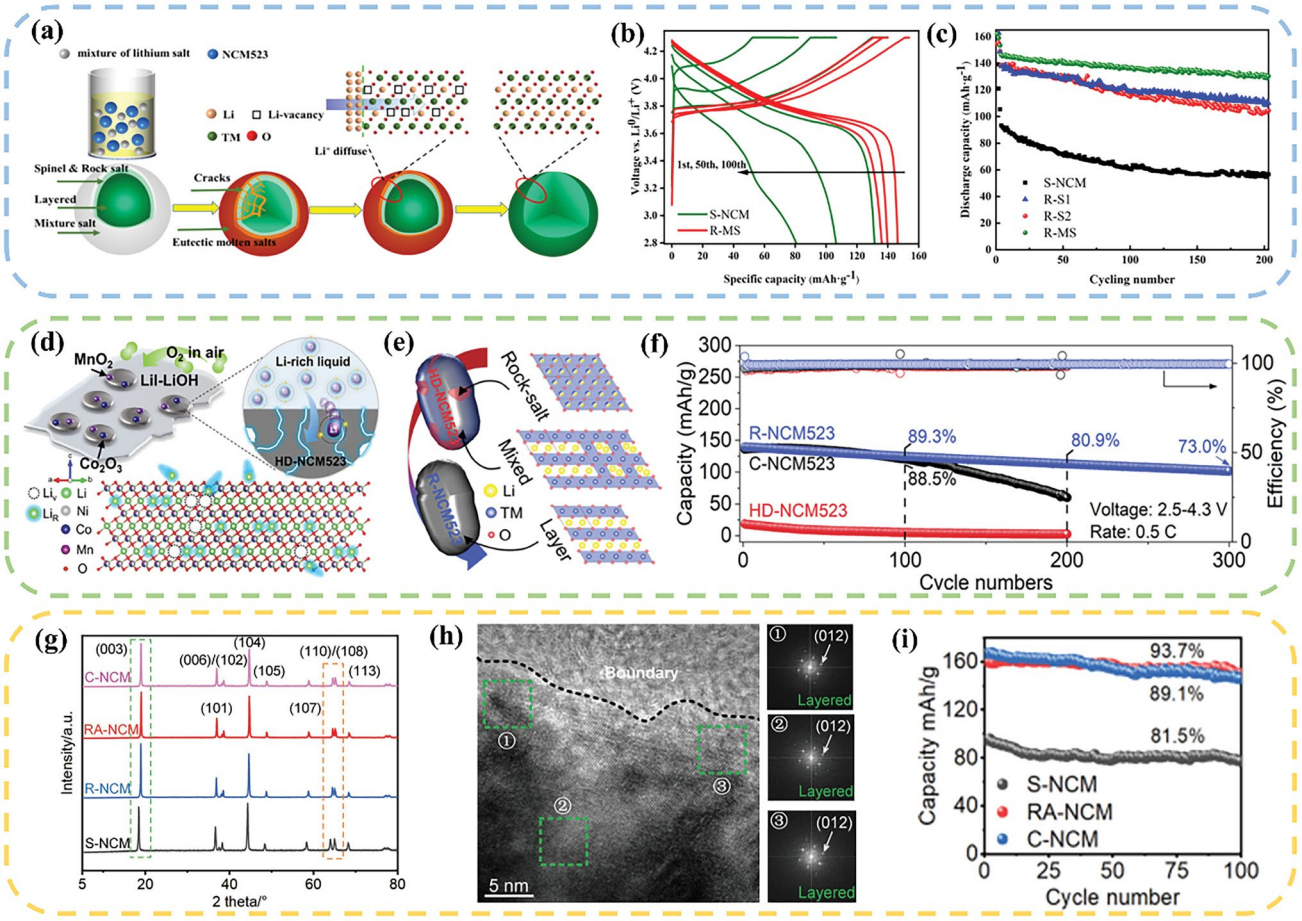
**a** Re-lithiation process by eutectic molten salt method. **b** Voltage-capacity curves of S–NCM and R–MS; **c** Cycle performance of S–NCM and all regenerated materials [127]. Copyright 2020, American Chemical Society. **d** Schematic diagram of direct recovery of HD–NCM523. **e** Schematic of the phase differences of HD–NCM523 and R–NCM523. **f** Cycling performance of HD–NCM523, R–NCM523, and C–NCM523 at 0.5 C [128]. Copyright 2020, American Chemical Society. **g** XRD patterns of S–NCM, R–NCM, RA–NCM, and C–NCM samples. **h** HRTEM image of recycled material, the image on the right corresponds to the FFT image of the corresponding area in the dashed box. **i** Diagram of the cycling performance of S–NCM, RA–NCM and C–NCM at 0.5 C [48]. Copyright 2022, WILEY–VCH Verlag

Jiang et al. [127] used LiOH–Li_2_CO_3_ molten salt as the lithium source to directly regenerate NCM523 at 440 °C. The Li^+^ diffused from the surface and filled into the lithium vacancies, which can inhibit the mixing of cations and transform the surface rock–salt phase and mixed phase to a well-structured layered phase (Fig. 7a). After 100 cycles, the discharge voltage of the reconstructed material goes up, and the capacity retention rate remains as 89% after 200 cycles (Fig. 7b, c). Ma et al. [128] chose a LiI–LiOH molten salt which has the lowest melting point in the binary eutectic system. Considering the dissolution of transition metal elements during the cycling process, transition metal oxides, such as Co_2_O_3_ and MnO_2_, were extra added to compensate the lost transition metal elements (Fig. 7d). Through the combined effect of one-step heating method by using eutectic molten salt and transition metal oxides, the lost elements were replenished, and the structure was repaired (Fig. 7e). The regenerated NCM523 exhibited comparable electrochemical performance to the commercial sample (Fig. 7f). Qin et al. [48] incorporated CH_3_COOLi into LiNO_3_–LiOH mixture to form a ternary eutectic melt, further reducing the eutectic point. Due to the lower density and larger volume of the ternary molten salt, the required amount of lithium salt for the reaction can be cut down. The S–NCM changes from a mixture of rock salt, spinel and layered phase to a single layered structure (Fig. 7g, h). The reversible capacity of the regenerated cathode at 0.5 C was 160 mAh g^−1^, and the capacity retention rate after 100 cycles was 94% (Fig. 7i).

Compared to other methods, the eutectic molten salt method has been primarily used to the restoration of ternary materials. At present, researchers have attempted to apply this method to other materials. In a study by Yang et al. [65], alkaline LiOH–KOH–Li_2_CO_3_ molten salt was utilized to reclaim the LCO under the atmosphere of ambient air at 500 °C. The LiOH–KOH and Li_2_CO_3_ were served as oxidizing flux and lithium source, respectively, which facilitate the decomposition of impurities through the mechanism of dissolution and subsequent recrystallization. The solvent system enables effective oxidation and dissolution, creating a high-temperature environment for the decomposition of carbon and PVDF. The high-concentration Li^+^ diffuse onto the impurity surface, which further cause the formation of new LCO, while the cobalt oxide undergoes dissolution in the molten salt and subsequently reprecipitates in a form of LiCoO_2_. The discharge capacity of the discarded LiCoO_2_ can be restored from 68 to 145 mAh g^−1^, reaching a capacity level of commercial LCO cathode. Typically, the oxidizing environment during the molten salt process oxidizes Fe^2+^ in LFP to Fe^3+^, leading to the destruction of the material’s crystal structure. Liu et al. [129] proposed a low-temperature molten salt method combined with a reducing environment that can inhibit the oxidation of Fe^2+^ and regenerate LFP materials. In their study, they utilized lithium nitrate as the molten salt medium and lithium source, and sucrose as the reducing agent. The reaction was conducted at 300 °C for 2 h. Through the carbonization process, the lithium-deficient iron phosphate was reduced from a + 3 to a + 2 valence state, counteracting the oxidizing ability of lithium nitrate at high temperatures and creating a reducing reaction environment. Combined with short annealing, the lithium-deficient and structurally damaged LFP particles were supplemented compositionally and restored structurally. The capacity of the regenerated LFP was restored to 145 mAh g^−1^ at 0.5 C. Table 3 summarizes the experimental conditions and regeneration effects of current eutectic molten salt method.

**Table 3:** A summary of eutectic molten salt method

Type	Parameters	Performance	References
LFP	Reagent: LiNO_3_, FeC_2_O_4_, 10 wt.% Sucrose molten salt: 300 °C, 2 h, Ar	Initial discharge capacity: 145 mAh g^−1^; Current density: 0.5 C; Cycle number: 100; Capacity retention rate: 90%	[129]
LFP	Reagent: LiNO_3_, Sucrose molten salt: 300 °C, 0.5 h	Initial discharge capacity: 162 mAh g^−1^; Current density: 0.5 C; Cycle number: 500; Capacity retention rate: 90%	[130]
NCM	Reagent: Li_2_SO_4_·H_2_O–CH_3_COOLi, LiOH·H_2_O molten salt: 450 °C, 4 h 900 °C, 10 h	Initial discharge capacity: 172 mAh g^−1^; Current density: 1 C; Cycle number: 200; Capacity retention rate: 83%	[131]
NCM	Reagent: LiNO_3_–LiOH molten salt: 300 °C, 4 h	Initial discharge capacity: 150 mAh g^−1^; Current density: 1 C; Cycle number: 100; Capacity retention rate: 90%	[126]
NCM	Reagent: Li_2_CO_3_–LiOH molten salt: 440 °C, 5 h	Initial discharge capacity: 146 mAh g^−1^; Current density: 1 C; Cycle number: 200; Capacity retention rate: 89%	[127]
NCM	Reagent: LiI–LiOH, Co_2_O_3_, MnO_2_ molten salt: 200 °C, 4 h	Initial discharge capacity: 150 mAh g^−1^; Current density: 0.5 C Cycle number: 300; Capacity retention rate: 73%	[128]
NCM	Reagent: LiOH–LiNO_3_–CH_3_COOLi molten salt: 400 °C, 4 h	Initial discharge capacity: 150 mAh g^−1^; Current density: 0.5 C; Cycle number: 100; Capacity retention rate: 94%	[48]
LCO	Reagent: LiOH–KOH molten salt: 300 °C, 8 h	Initial discharge capacity: 149 mAh g^−1^; Current density: 0.2 C; Cycle number: 200; Capacity retention rate: 93%	[132]
LCO	Reagent: LiOH–KOH–Li_2_CO_3_ molten salt: 500 °C, 8 h	Initial discharge capacity: 145 mAh g^−1^; Current density: 0.2 C; Cycle number: 200; Capacity retention rate: 93%	[65]

### Electrochemical Method

The electrochemical regeneration involves the use of spent cathodes as the working electrode and a lithium-containing solution as the electrolyte. By applying a constant current to the cathode material, Li^+^ can be driven by the electrons in the external circuit to migrate to the vacant Li sites, which effectively reduces the migration activation energy. When the energy in the external circuit exceeds the migration activation energy, the Li^+^ in the electrolyte enter the lithium vacancies in the cathode material. Combined with a short-term annealing treatment, the crystal structure can be further restored, finally achieving the cathode regeneration [133, 134].

**Fig. 8:**
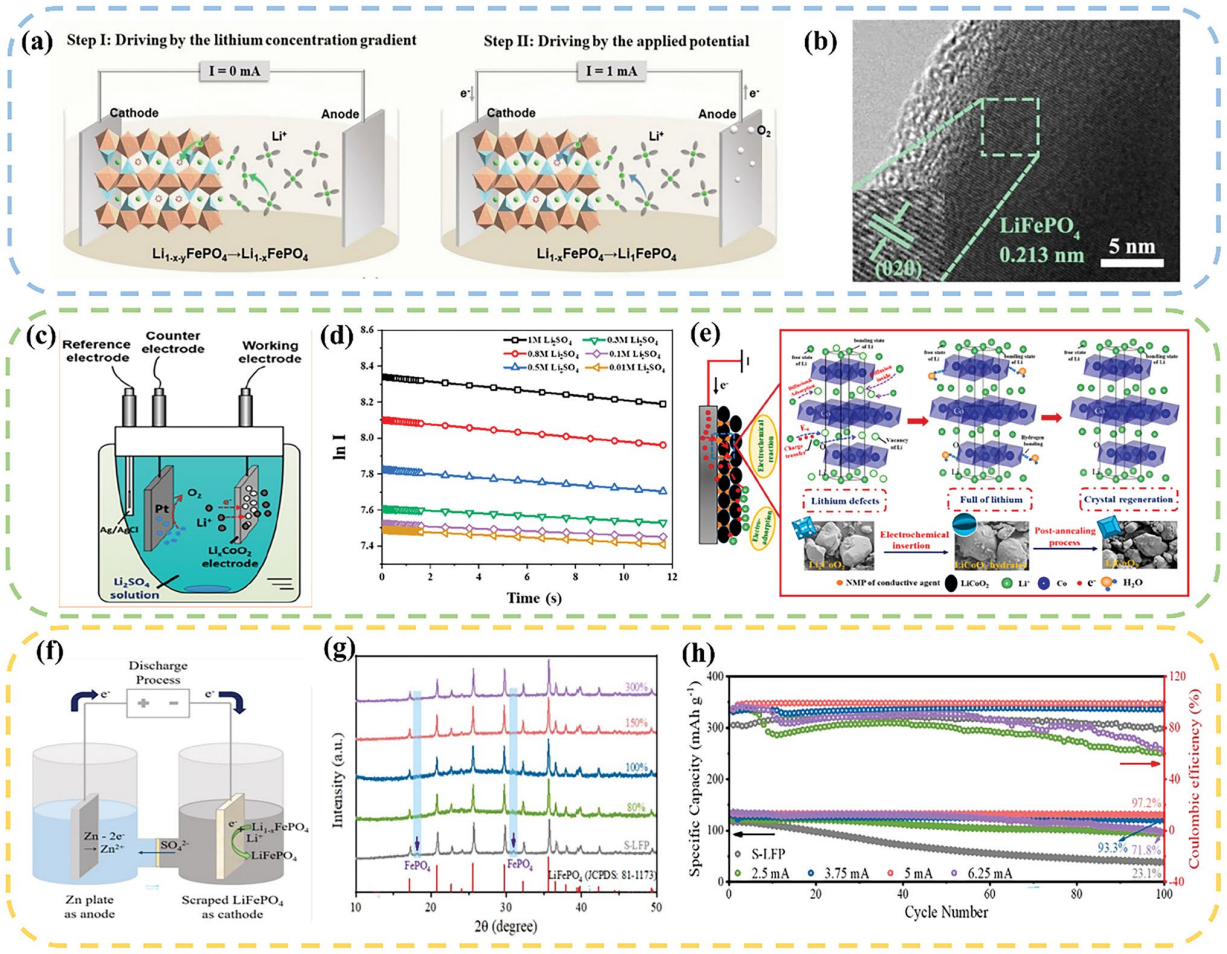
**a** Schematic diagram of the regeneration of S–LFP by output current lithiation. Step I: without output current lithiation process, Step II: with external output current lithiation process. **b** HRTEM image of R–LFP[135]. Copyright 2022, RSC Publishing. **c** Diagram of the electrochemical reaction device; **d** ln *I*–*t* curve of cathode electrode with different Li^+^ concentrations; **e** Proposed mechanism of direct regeneration of LiCoO_2_ materials by electrochemical insertion of Li^+^ into the waste Li_*x*_CoO_2_ electrode [136]. Copyright 2020, American Chemical Society. **f** Schematic diagram of re–lithiation method. **g** XRD spectra of recovered S–LFP and R–LFP under different TIA. **h** Cycling performance of S–LFP and R–LFP at 1 C (1 C = 170 mAh·g^−1^) under different TIA conditions [137]. Copyright 2022, RSC Publishing

Peng et al. [135] employed a spontaneous and electrically driven collaborative approach to achieve the direct regeneration of LFP. They utilized a spent cathode as the working electrode, Ag/AgCl electrode as the reference electrode, and Pt mesh as the counter electrode, which establish a three-electrode system. In a Li_2_SO_4_ electrolyte, without applying a current, the spontaneous migration of Li^+^ occurs by the concentration difference, resulting in the formation of Li_1–*x*_FePO_4_. Driven by the current, Li^+^ and Li_1–x_FePO_4_ can combine together to form LFP (Fig. 8a, b). Compared to conventional current-driven methods, this approach effectively shortens the reaction time, and the regenerated LFP delivers a capacity retention rate of 95% after 500 cycles. In a similar way, Zhang et al. [136] employed a comparable method to directly regenerate the LCO cathode (Fig. 8c). Under the condition of high-concentration electrolyte, the redox process was primarily governed by the charge transfer behavior. Conversely, in a low-concentration electrolyte, the process was predominantly influenced by Li^+^ diffusion behavior (Fig. 8d). Higher electrolyte concentration and current density prove advantageous to accelerating the reduction of Li^+^ (Fig. 8e).

Generally, the regenerated materials necessitate an annealing process to restore their crystalline structure. Zhou et al. [137] developed an electrochemical regeneration method that eliminates the need for subsequent annealing. In an H–type electrolytic cell, a spent LFP suspension was used as the cathode, and a zinc plate served as the anode. The two electrodes were separated by an anion exchange membrane (Fig. 8f). The application of an electric field facilitated the insertion of Li^+^ from the electrolyte into the S–LFP structure, inducing the transformation of FePO_4_ to LiFePO_4_ and ultimately regenerating the LFP (Fig. 8g). When subjected to the optimal condition (applied current was set as 5 mA), the R–LFP showed a specific capacity of 134 mAh g^−1^ after 300 cycles, retaining 86% of its original capacity. (Fig. 8h). Table 4 summarizes the experimental conditions and regeneration effects of current electrochemical method. Currently, electrochemical regeneration methods are mainly applied to the direct regeneration of LFP and LCO materials, and no literature has been reported on NCM materials.

**Table 4:** A summary of electrochemical method

Type	Parameters	Performance	References
LFP	Cathodic current: 0.40 mA cm^−2^	Initial discharge capacity: 154 mAh g^−1^; Current density: 1 C; Cycle number: 300; Capacity retention rate: 91%	[138]
LFP	Cathodic current: 1 mA	Initial discharge capacity: 135 mAh g^−1^; Current density: 1 C; Cycle number: 500; Capacity retention rate: 95%	[135]
LFP	Cathodic current: 5 mA	Initial discharge capacity: 134 mAh g^−1^; Current density: 1 C; Cycle number: 300; Capacity retention rate: 85%	[137]
LFP	Voltage: 1 V	Initial discharge capacity: 137 mAh g^−1^; Current density: 1 C; Cycle number: 300; Capacity retention rate: 95%	[39]
LCO	Cathodic current: − 0.42 mA cm^−2^	Initial discharge capacity: 136 mAh g^−1^; Current density: 1 C; Cycle number: 200; Capacity retention rate: 99%	[136]

### Chemical Lithiation Method

The chemical lithiation method is less commonly used in the direct regeneration process. However, compared to other direct regeneration methods, chemical lithiation regeneration consumes much shorter reaction time.

**Fig. 9:**
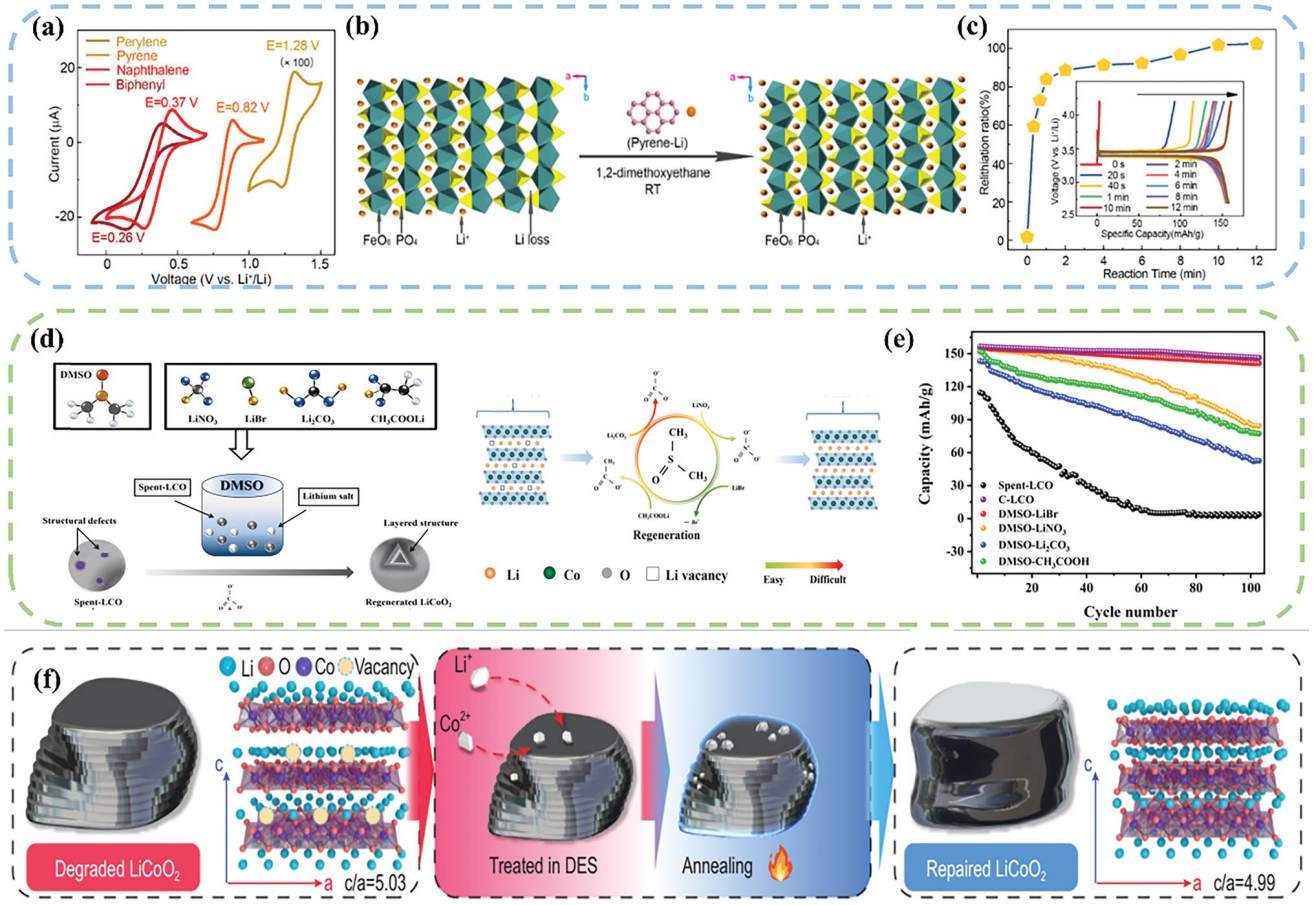
**a** Cyclic voltammetry curves tested in biphenyl/DME, naphthalene/DME, pyrene/DME, and perylene/DME solutions at a scan rate of 50 mV s^−1^. **b** Schematic diagram of the chemical recovery of waste LFP cathode. **c** Evolution of the reduction ratios with different reaction times. The inset shows the initial charge/discharge profiles of the regenerated LFP cathode after different lithiation times [139]. Copyright 2018, RSC Publishing. **d** Schematic diagram of the autoxidation reaction mechanism on waste LCO cathode. **e** Cycling performance tested in different systems [140]. Copyright 2022, Elsevier. **f** Schematic diagram of the repair mechanism on degraded LCO [141]. Copyright 2022, China Science Publishing & Media Ltd

Wu et al. [139] proposed a thermodynamically spontaneous Li^+^–electron synergistic radical oxidation–reduction process by using a reductive polycyclic aryl lithium compound with tunable potential as the reductant and Li^+^ donor. Since the pyridine–lithium complex (Py–Li) possesses a suitable potential of 0.82 V compared to Li^+^/Li, which is significantly higher than the decomposition potential of LFP (0.59 V vs. Li^+^/Li), it can effectively reduce the Li^+^ defects in the LFP lattice without damaging its crystal structure (Fig. 9a). It is the potential difference between the cathode material and the aromatic lithium reagent that drives the electrons transferring from the pyridyl radical anion (Py*) to Li_1–*x*_FePO_4_ and allows the diffusion of Li^+^ into the bulk lattice (Fig. 9b). The entire chemical reduction process can be carried out at ambient temperature and pressure, and the reaction can be completed in just 10 min (Fig. 9c). After the lithiumation reaction is completed, the introduction of metallic Li into the solution can reverse the Py neutral molecules back to Py* radical anions, which realizes the recycling of the reagent. Fei et al. [140] developed a novel self-oxidation system using lithium bromide as the lithium source, dimethyl sulfoxide (DMSO) as the solvent and oxygen donor. The ionic nature of DMSO facilitates the strong interaction between ions and its inherent volatility provides an oxidative atmosphere for the regeneration process. Additionally, due to its nucleophilic property, the Li^+^ in LiBr are released, and the co-solvent provides the high charge flux medium for the transport of Li^+^ and O^2–^, enabling the self-oxidation of transition metal elements (Fig. 9d). The cracks and impurities (such as Co_3_O_4_) on the surface of spent materials are repaired at a low temperature and environmental pressure. The capacity retention rate of the regenerated LCO material reaches 91%, which is slightly higher than that of commercial LCO. (Fig. 9e).

Most direct regeneration methods focus on lithium replenishment, and only limited researches consider the supplementation of dissolved transition metal elements. According to the previous work [141], the cobalt dissolution significantly affects the capacity decay and cycling stability of the LCO cathode. In order to fully regenerate the LCO under environmental pressure, it is crucial to prioritize the supply of Co^2+^. The urea molecule shows a stronger affinity to Co^2+^ as compared to Li^+^, suggesting the preferential transfer of Co^2+^ from the solution to the spent LCO. In this regard, a deep eutectic solvent (DES) LiCl–CH_4_N_2_O (urea) was invented for the regeneration of spent LCO, which can be used under atmospheric condition. During the regeneration process, partial Li^+^ and Co^2+^ will transfer at the material surface, and then gradually diffuse and occupy the lattice vacancies. In combination with the annealing step, the atomic rearrangement of Li and Co takes place, leading to the conversion of the spinel phase into a layered phase (Fig. 9f). This method demonstrates environmental and economical as it reduces the energy consumption by 37% and lowers the greenhouse gas emissions by 35% when compared to conventional LCO production process. Table 5 summarizes the experimental conditions and regeneration effects of current chemical lithiation method.

**Table 5:** A summary of chemical lithiation method

Type	Parameters	Performance	References
LFP	Reagent: Py–Li, NO_2_BF_4_, DME	Initial discharge capacity: 156 mAh g^−1^; Current density: 0.5 C; Cycle number: 150; Capacity retention rate: 77%	[139]
LFP	Reagent: LiI, acetonitrile	Initial discharge capacity: 140 mAh g^−1^; Current density: 0.1 C	[142]
NCM	Reagent: 3, 5–di–*tert*–butyl–*o*–benzoquinone (DTBQ), dimethoxyethane (DME)	Initial discharge capacity: 183 mAh g^−1^	[143]
NCM	Reagent: LiBr	Initial discharge capacity: 160 mAh g^−1^; Current density: 0.1 C; Cycle number: 100; Capacity retention rate: 85%	[144]
LCO	Reagent: DMSO–LiBr	Initial discharge capacity: 155 mAh g^−1^; Current density: 1 C; Cycle number: 100; Capacity retention rate: 91%	[140]
LCO	Reagent: LiCl–CH_4_N_2_O	Initial discharge capacity: 130 mAh g^−1^; Current density: 0.5 C; Cycle number: 100; Capacity retention rate: 90%	[141]
LCO	Reagent: betaine, ethylene glycol lithium, urea	Initial discharge capacity: 210 mAh g^−1^; Current density: 1 C; Cycle number: 200; Capacity retention rate: 87%	[145]

Solid-state sintering is a widely used direct regeneration method, offering a simple operation that allows for material lithium replenishment and structural repair in one step. It enables direct regeneration of cathode materials with excellent performance. However, this regeneration process requires a long time in a high-temperature environment and consumes a significant amount of energy. Moreover, the use of solid-phase lithium source can result in material inhomogeneity due to poor solid–solid contact, which in turn affects the subsequent material regeneration process. Additionally, the amount of lithium loss varies between each batch of material, necessitating accurate calculations for lithium replenishment before each regeneration. This undoubtedly increases the workload in the recycling process and reduces the efficiency of the regeneration.

Hydrothermal regeneration method has a relatively low reaction temperature (< 250 °C) and low energy consumption. The liquid lithium source facilitates the diffusion of Li^+^ in the material and effectively solves the problem of uneven mixing of lithium source and material. Meanwhile, lithium supplementation does not require precise chemical calculations due to the improved reaction kinetics. However, the hydrothermal reaction necessitates a specialized vessel and a specific pressure, and the liquid lithium source cannot be recycled, leading to increased costs. The hydrothermal method alone can only realize the supplementation of the missing elements, and subsequently needs to be combined with short-time annealing to repair the crystal structure of the material.

The eutectic molten salt method utilizes eutectic molten salt as a lithium source, effectively reducing the reaction temperature. This method offers a clear advantage over solid-phase sintering due to its lower reaction temperature and shorter reaction time, resulting in lower energy consumption. The melting of the molten salt creates a lithium-rich environment, enhancing the diffusion kinetics of Li^+^ at the interface. As a result, there is no need to calculate the amount of lithium loss. Moreover, the eutectic molten salt method efficiently replenishes elemental losses and effectively repairs structural damage in the material, leading to a high regeneration efficiency. However, it’s important to note that current studies primarily focus on the repair and regeneration of NCM materials. There are fewer reports on the regeneration of LFP and LCO materials, and the applicability to different materials has yet to be explored.

Electrochemical regeneration utilizes a potential difference to promote the replenishment of Li^+^ with low energy consumption and cost. The efficiency of lithium replenishment can be further enhanced by adjusting factors such as the concentration of the lithium solution and the magnitude of the current. However, subsequent annealing treatments are required to repair the material structure. Unlike other methods, the electrochemical method allows for the direct insertion of the disassembled cathode sheet into the lithium-containing solution as an electrode. However, this method places high demands on the disassembly of materials, and achieving satisfactory regeneration results for highly degraded materials becomes challenging. Moreover, the regeneration efficiency of this method is comparatively low. Additionally, the electrochemical method has limitations in terms of material selection.

Chemical lithiation regeneration involves the use of lithium-based chemical reagents at room temperature and pressure. It utilizes the chemical potential to achieve lithium supplementation. However, for the recovery of the material structure, additional processing steps are required, resulting in low regeneration efficiency. It’s important to note that most of the chemical reagents used are volatile organic solvents. While these solvents can be recycled multiple times, caution must be exercised regarding their potential harm to human health and the environment during usage.

Table 6 compares the five direct regeneration methods from four aspects: cost, energy consumption, pollution emissions, and regeneration efficiency.

**Table 6:**
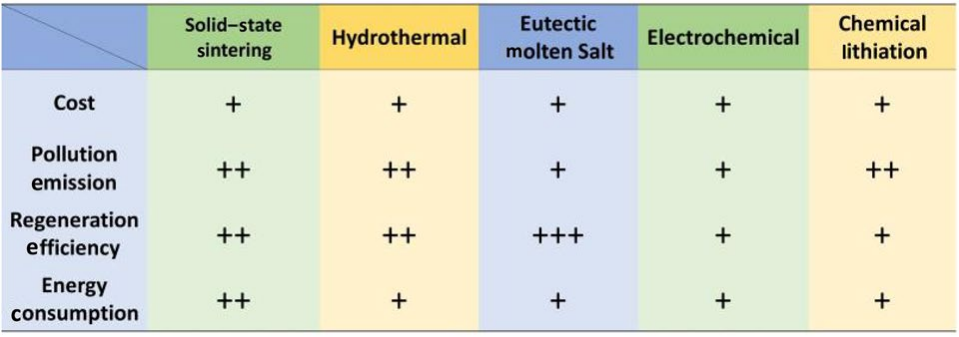
A comparison of different direct regeneration methods

## Production Practice

So far, most researches on the direct regeneration primarily concentrate on laboratory investigations. The aforementioned regeneration methods have yielded satisfactory restorative outcomes at the laboratory level, where the morphology, structure and electrochemical performance of the repaired materials have been restored to, or even exceeded, their original levels. However, the related works on the application of direct regeneration in actual production are extremely rare. Due to the exponential rise in retired batteries, numerous enterprises have augmented their investments in battery recycling. Wuhan Rikomay New Energy Co., Ltd. has developed a set of technologies and equipments for direct regeneration of spent LFP, and become the first battery recycling company to achieve large-scale mass production using physical methods and short processes. Bump Recycling, a subsidiary of CATL, has spearheaded the core technology of “targeted recycling” for spent batteries, boasting an exceptional total recovery rate of core metallic materials (over 99%). Tianjin Sai De Mei New Energy Technology Co., Ltd. has developed a magnetic separation combined with color sorting technology, enabling complete separation of cathode and anode components. Through precise identification of missing elements and research on deformation patterns, this company has exploited technologies and equipments to control the element compensation and concentration uniformity. The development of these advanced technologies promotes the growth of battery regeneration industries and facilitates the industrial application of direct regeneration methods. In this section, we plan to introduce the practical application of direct regeneration in industrial production by using Tianjin Sai De Mei’s annual production line of 10,000 tons for regenerating spent ternary materials as an example.

The recycling process mainly consists of the following steps. Firstly, the individual cells with a voltage exceeding 3 V have to be discharged. Subsequently, a precise dismantling procedure is implemented to segregate the cathode, anode and additional constituents. After the cathode electrode plate is pulverized, it is separated from the Al foil to obtain the spent cathode powder. The regenerated cathode powder can be finally obtained by composition regulation and solid-state sintering.

### Pretreatment

**Fig. 10:**
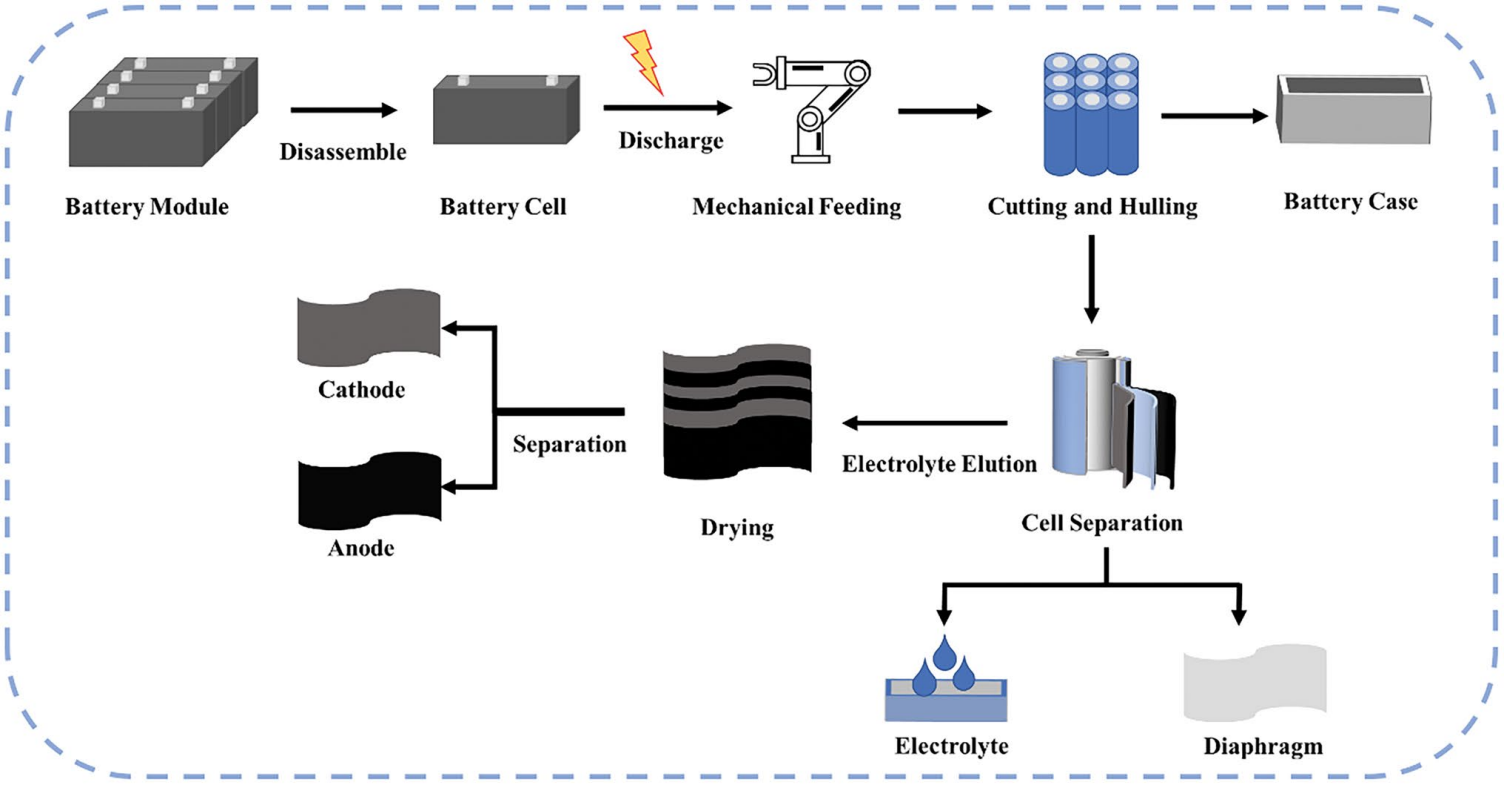
Precise dismantling process of spent ternary lithium‒ion battery monoblock

The spent LIBs firstly go through a screening process, and then disassembled into individual battery packs. Afterward, each of the acquired battery undergoes testing to assess its remaining capacity and determine its suitability for secondary reuse. Batteries that have less than 80% of their initial capacity and do not hold any secondary reuse value, will undergo a precise dismantling process as outlined in Fig. 10. To ensure the safety of subsequent processing steps, the individual batteries are discharged using a constant potential discharge device, which reduces the voltage to a range between 1.50 and 2.75 V. The discharged battery packs are separated into outer casings and inner cores through cutting, and the internal electrolyte is removed using an elution solution. After undergoing drying, the electrode sheets are sorted. The cathode materials obtained from sorting are then crushed, resulting in a mixed powder of cathode materials with varying particle sizes and Al foil. By exploiting the disparity in particle density, these materials are separated using vibrating sieves with mesh sizes ranging from 50 to 300 mesh. Finally, the obtained cathode powder enters into the direct regeneration section.

### Direct Regeneration

**Fig. 11:**
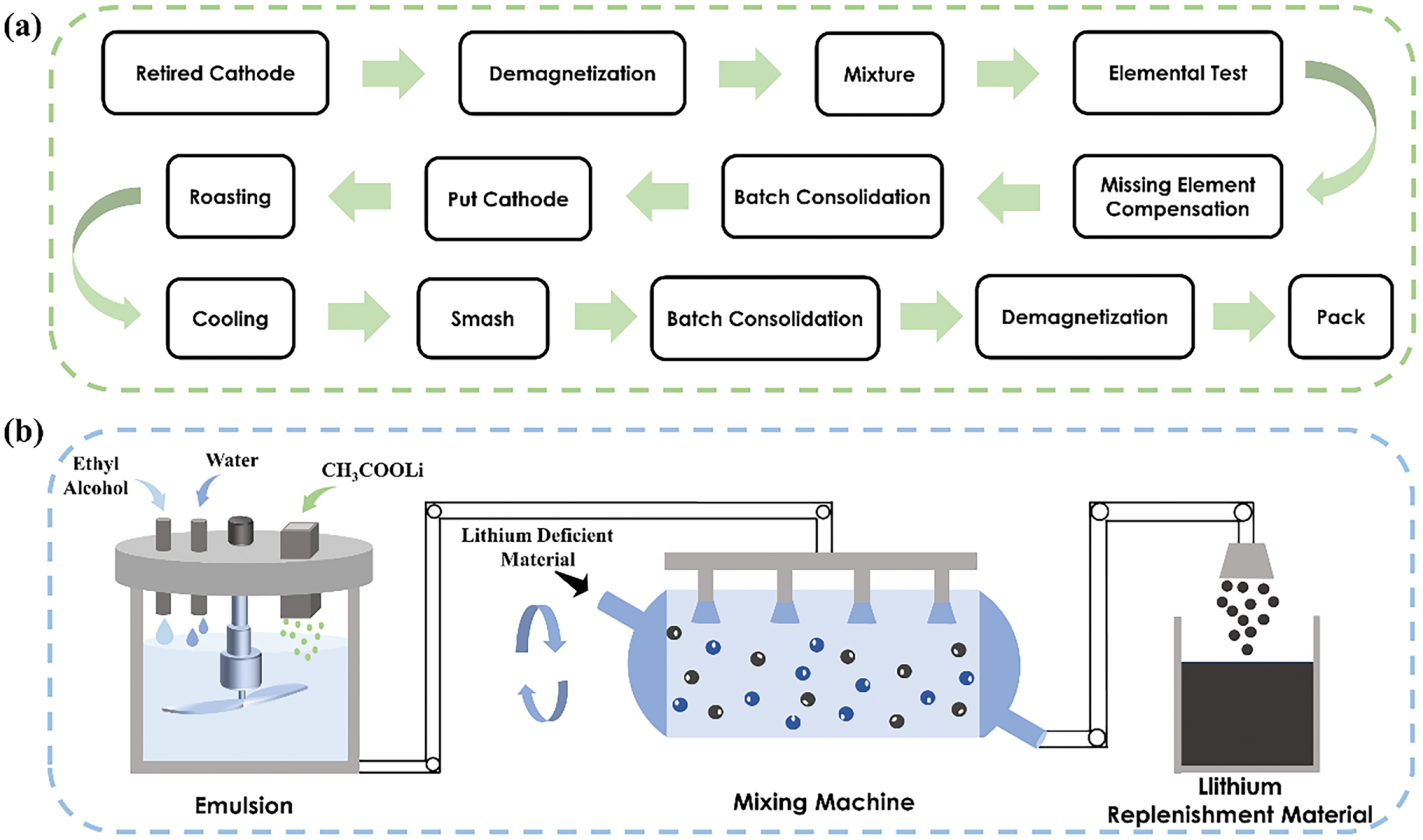
**a** Direct regeneration process of spent lithium-ion battery ternary cathode material. **b** Compensation process of missing element

The direct regeneration process includes mixing, testing, compensating missing elements, and high-temperature sintering, as depicted in Fig. 11a. In practical production, a small amount of magnetic impurities may be introduced into the cathode powder due to environmental control issues. To address this problem, a demagnetizer is employed in the first step to remove the magnetic substances mixed in the powder. The treated cathode powder is then mixed by a spiral ribbon blender to ensure the homogeneity of the material mixture and stabilize the regenerative effect across different batches. Elemental measurements are conducted on the mixed materials to ascertain the mass percentages of key elements (Li, Ni, Co, Mn, Al) and identify the types of materials to be recovered. Considering the propensity of lithium to volatilize during high-temperature calcination, the amount of lithium added during the mixing stage is calculated based on the ratio of (Ni + Co + Mn):Li = 1:1.05.

The process of compensating missing elements is depicted in Fig. 11b. A mixture is prepared by combining pure water and anhydrous ethanol in a ratio of 9:1. Subsequently, anhydrous lithium acetate is added and thoroughly stirred to form an emulsion. The lithium source is then spray-mixed with the cathode material powder, ensuring an equal distribution of the lithium source and cathode powder throughout the spraying process via continuous stirring. The materials are uniformly conveyed into a continuous and controllable atmosphere furnace via a conveyor belt for solid-state sintering under an O_2_ atmosphere. The furnace is divided into heating, constant temperature and cooling stages, encompassing 18 temperature control zones in total, with the temperature of each zone maintained between 100 and 950 °C. After regeneration, the cooled materials are crushed and enter the grading chamber. Separated fine particles that meet the particle size requirements are collected, mixed, demagnetized and subsequently packaged. The regenerated NCM523 demonstrates an initial discharge capacity of 140 mAh g^−1^ at a current rate of 0.1 C, closely approaching the theoretical capacity of 150 mAh g^−1^. These results are comparable to the restoration effect achieved at the laboratory scale, thereby providing ample evidence to support the viability of employing the direct regeneration method in large-scale industrial applications.

Among the five direct regeneration methods, solid-state sintering is easy to operate and can achieve elemental replenishment and structural recovery in one step. It is applicable to different types of materials and is more suitable for large-scale industrialized production situations. However, when facing the situation of a large quantity and mixing of different batches of materials, the accurate calculation of lithium loss and achieving homogeneous mixing of materials and lithium source become problems that need to be solved during practical operation. The hydrothermal method eliminates the need for calculating the amount of lithium supplementation and solves the difficulty of mixing materials with the lithium source. However, its application in large-scale industrialized production is limited due to the requirement of a specific container and the high-pressure working environment, which pose safety concerns. Eutectic molten salt, electrochemical, and chemical lithiation methods can effectively supplement missing elements and restore the structure, especially when combined with subsequent annealing. However, their limitations raise concerns about whether they can meet the requirements of large-scale industrial applications. For example, the eutectic molten salt method has shown promising repair effects for NCM, but reports on its efficacy for LFP and LCO are relatively scarce. It is worth exploring whether it can be universally applied to different material types. Electrochemical regeneration is more suitable for repairing materials with low levels of degradation and minimal structural damage, while achieving satisfactory repair effects for highly degraded materials proves challenging. The chemical lithiation process involves the use of organic chemical reagents, and finding ways to efficiently use and environmentally recycle these reagents is a future direction that requires attention.

## Conclusion and Outlook

**Fig. 12:**
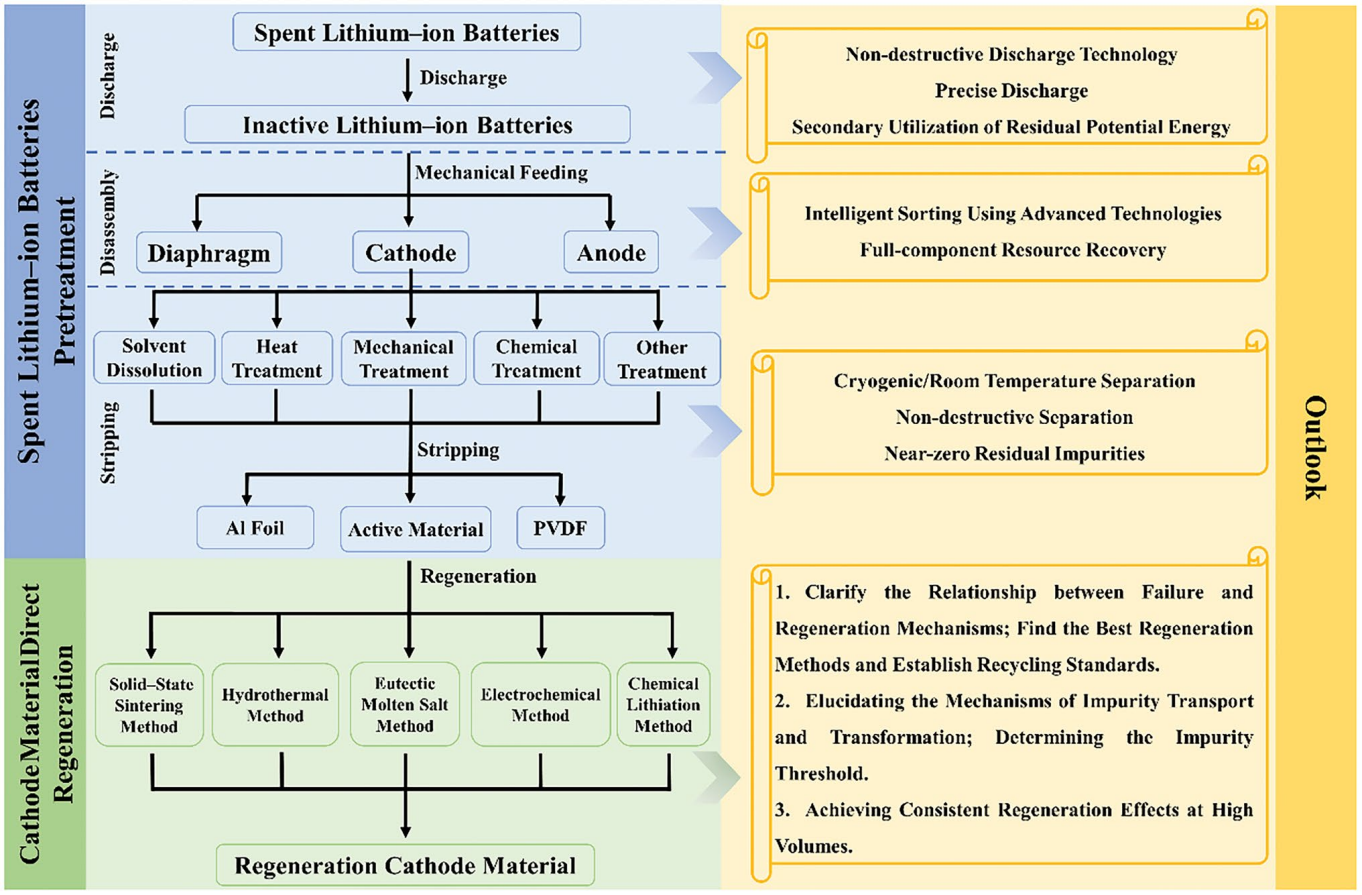
Direct regeneration production technology routes and prospects

In this article, we start by exploring the failure mechanisms of three representative battery cathodes, LFP, NCM and LCO. The primary reason for the failure of LFP is the presence of lithium vacancies (Li_V_) and Li/Fe antisite defects, the NCM capacity attenuation is mainly due to the structural change caused by the loss of elements, and the structural collapse and the formation of unfavorable phase interface are the main reasons for the deterioration of LCO performance. Each material manifests distinctive failure reasons, where those experiencing minimal structural damage are deemed more suitable for direct regeneration. Moreover, the quality of the pretreatment stage assumes a critical role in determining the effectiveness of material regeneration. We accentuate the research progress of direct regeneration methods for cathode materials, and exemplify the application of direct regeneration technology in the battery recycling industry through the Tianjin Sai De Mei direct regeneration production line. Building upon this foundation, Fig. 12 portrays a foresight into the future large-scale industrial implementation of direct regeneration methods.

1. In the future, nondestructive discharge technology can be developed for large-scale industrialized production. This technology involves discharging decommissioned batteries by utilizing advanced instruments to achieve precise control over the discharging process. The benefits include shorter discharging time, improved operational efficiency, reduced corrosion of electrode materials during discharging, and prevention of possible structural damage. In addition, methods for the secondary utilization of residual energy within the battery can be explored, such as harnessing the remaining energy for material restoration purposes.
2. Currently, most enterprises heavily rely on manual dismantling of used batteries for accurate component separation in production. However, this method is inefficient and costly. During the disassembly process, a portion of the electrolyte will volatilize, generating toxic and harmful gases that pose health hazards. In future research, advanced technologies such as artificial intelligence and visual imaging can be developed to replace traditional manual work with more efficient methods. This will enable intelligent and highly efficient precision dismantling processes, which are better suited for mass production scenarios. Additionally, other components, such as battery shells obtained from dismantling, can be utilized for resource recycling, thus achieving the complete recycling of all retired lithium battery components.
3. In industrial production, most companies utilize crushing and sorting techniques to recover cathode powder from batteries. However, a certain amount of impurities, such as Al, Fe, and Cu, inevitably remain in the recovery process. In the future, we can further optimize the separation methods between the material and the aluminum foil. This can be achieved by developing low-temperature or room-temperature separation technology to reduce energy consumption. Additionally, we can focus on developing nondestructive separation technology to improve the separation rate between the cathode material and the collector. These advancements aim to realize near–zero residue of impurities in cathode materials.
4. In future research, it is necessary to clarify the relationship between the failure mechanism of different types of materials and the regeneration mechanism. For decommissioned materials with varying degrees of degradation, optimization of the regeneration conditions should be based on the characteristics of the waste materials. The goal is to find an optimal regeneration method that achieves low energy consumption, low cost, and high efficiency. Additionally, it is important to establish recycling standards and provide support for the large-scale industrial application of direct regeneration methods.
5. Current laboratory studies have shown that the presence of small amounts of impurities can improve the electrochemical properties of regenerated materials. In the future, further exploration of the effects of impurities on the repair and regeneration process is necessary. This will help elucidate the migration and transformation mechanisms of impurity elements and determine the threshold value for their presence. Scale–up experiments will be conducted to evaluate the amplification of impurity effects in large-scale applications. These experiments will provide guidance for practical production.
6. Currently, direct regeneration is primarily based on laboratory research and often yields better results in small-scale experiments. However, in actual production, variations in production conditions and material batches lead to differences in the stability of regenerated materials and the consistency of regeneration results. It becomes challenging to achieve the same level of regeneration as observed in laboratory-scale experiments. In future research, it is recommended to conduct experiments above the pilot scale at the production site. This will allow for the optimization of process flow and adjustment of experimental parameters, ultimately enhancing the stability and consistency of products during large-scale production.
