# Correction: Direct Regeneration of Spent Lithium-Ion Battery Cathodes: From Theoretical Study to Production Practice

**DOI:** 10.1007/s40820-024-01459-5

**Published:** 2024-06-25

**Authors:** Meiting Huang, Mei Wang, Liming Yang, Zhihao Wang, Haoxuan Yu, Kechun Chen, Fei Han, Liang Chen, Chenxi Xu, Lihua Wang, Penghui Shao, Xubiao Luo

**Affiliations:** 1 National–Local Joint Engineering Research Center of Heavy Metals Pollutants Control and Resource Utilization, Nanchang Hangkong University, Nanchang, 330063 People’s Republic of China; 2 Key Laboratory of Hunan Province for Advanced Carbon–based Functional Materials, School of Chemistry and Chemical Engineering, Hunan Institute of Science and Technology, Yueyang, 414006 People’s Republic of China; 3 College of Materials Science and Engineering, Central South University of Forestry and Technology, Changsha, 410004 People’s Republic of China; 4 School of Life Science, Jinggangshan University, Ji’an, 343009 People’s Republic of China

**Correction to: Nano-Micro Lett. (2024) 16:207** 10.1007/s40820-024-01434-0

The Highlights session of the article unfortunately was taken falsely from another manuscript. The correct Highlights session is now in place.

The correct is:

- Analyze the primary causes of cathode failure in three representative batteries, illustrating their underlying regeneration mechanism.
- The latest research status of direct regeneration of spent lithium–ion batteries was reviewed and summarized in focus.
- The application examples of direct regeneration technology in production practice are introduced for the first time, and the problems exposed in the initial stage of industrialization were revealed.

The original article has been corrected.

